# Risk prediction of covid-19 related death or hospital admission in adults testing positive for SARS-CoV-2 infection during the omicron wave in England (QCOVID4): cohort study

**DOI:** 10.1136/bmj-2022-072976

**Published:** 2023-06-21

**Authors:** Julia Hippisley-Cox, Kamlesh Khunti, Aziz Sheikh, Jonathan S Nguyen-Van-Tam, Carol A C Coupland

**Affiliations:** 1 Nuffield Department of Primary Health Care Sciences, University of Oxford, Oxford, UK; 2 Diabetes Research Centre, University of Leicester, Leicester, UK; 3 Usher Institute, University of Edinburgh, Edinburgh, UK; 4 Centre for Academic Primary Care, School of Medicine, University of Nottingham, Nottingham, UK

## Abstract

**Objectives:**

To derive and validate risk prediction algorithms (QCOVID4) to estimate the risk of covid-19 related death and hospital admission in people with a positive SARS-CoV-2 test result during the period when the omicron variant of the virus was predominant in England, and to evaluate performance compared with a high risk cohort from NHS Digital.

**Design:**

Cohort study.

**Setting:**

QResearch database linked to English national data on covid-19 vaccinations, SARS-CoV-2 test results, hospital admissions, and cancer and mortality data, 11 December 2021 to 31 March 2022, with follow-up to 30 June 2022.

**Participants:**

1.3 million adults in the derivation cohort and 0.15 million adults in the validation cohort, aged 18-100 years, with a positive test result for SARS-CoV-2 infection.

**Main outcome measures:**

Primary outcome was covid-19 related death and secondary outcome was hospital admission for covid-19. Risk equations with predictor variables were derived from models fitted in the derivation cohort. Performance was evaluated in a separate validation cohort.

**Results:**

Of 1 297 922 people with a positive test result for SARS-CoV-2 infection in the derivation cohort, 18 756 (1.5%) had a covid-19 related hospital admission and 3878 (0.3%) had a covid-19 related death during follow-up. The final QCOVID4 models included age, deprivation score and a range of health and sociodemographic factors, number of covid-19 vaccinations, and previous SARS-CoV-2 infection. The risk of death related to covid-19 was lower among those who had received a covid-19 vaccine, with evidence of a dose-response relation (42% risk reduction associated with one vaccine dose and 92% reduction with four or more doses in men). Previous SARS-CoV-2 infection was associated with a reduction in the risk of covid-19 related death (49% reduction in men). The QCOVID4 algorithm for covid-19 explained 76.0% (95% confidence interval 73.9% to 78.2%) of the variation in time to covid-19 related death in men with a D statistic of 3.65 (3.43 to 3.86) and Harrell’s C statistic of 0.970 (0.962 to 0.979). Results were similar for women. QCOVID4 was well calibrated. QCOVID4 was substantially more efficient than the NHS Digital algorithm for correctly identifying patients at high risk of covid-19 related death. Of the 461 covid-19 related deaths in the validation cohort, 333 (72.2%) were in the QCOVID4 high risk group and 95 (20.6%) in the NHS Digital high risk group.

**Conclusion:**

The QCOVID4 risk algorithm, modelled from data during the period when the omicron variant of the SARS-CoV-2 virus was predominant in England, now includes vaccination dose and previous SARS-CoV-2 infection, and predicted covid-19 related death among people with a positive test result. QCOVID4 more accurately identified individuals at the highest levels of absolute risk for targeted interventions than the approach adopted by NHS Digital. QCOVID4 performed well and could be used for targeting treatments for covid-19 disease.

## Introduction

During the first waves of the covid-19 pandemic, before the introduction of vaccines, identifying people at highest risk of severe covid-19 outcomes if they were to become infected with the SARS-CoV-2 virus was necessary. The QCOVID risk assessment tool for predicting risk of covid-19 related death or hospital admission based on individual characteristics was developed,^1^ independently externally validated in England,^2^ Wales,^3^ and Scotland,^4^ and was found to have performed well in identifying those at high risk of severe outcomes from covid-19. QCOVID was used in England in February 2021 to identify patients at high risk of severe covid-19 outcomes, adding another 1.5 million people to the national list of patients who required shielding. QCOVID was also used for prioritising people for vaccination across the UK (if they had not already been offered the vaccine based on their age or other risk classification).^5^ The QCOVID model was initially recalibrated during the first pandemic wave^1^ and then updated after the second and third waves of the pandemic to create two new versions of the model: QCOVID2, based on patients who were not vaccinated,^6^ and QCOVID3, based on patients who were partially vaccinated.^6^ These models accounted for changes that had occurred in the virus as well as the implementation of the vaccination programme.^6^

In December 2021 in the UK, a new wave of covid-19 infections with the omicron variant of the SARS-CoV-2 virus rapidly replaced high circulating levels of the previous delta variant. Although the omicron variant (BA.1) was associated with a lower risk of covid-19 related death than the delta variant,^7^ further mutations have occurred and concerns have been raised that covid-19 vaccines might become less effective. More treatments are likely to be needed to protect vulnerable individuals, such as antiviral agents and neutralising monoclonal antibodies.^8^ On 9 December 2021, neutralising monoclonal antibodies became available in the UK for high risk patients with symptoms of SARS-CoV-2 infection who did not require hospital admission.^9^ Neutralising monoclonal antibodies are a limited resource and hence have been targeted to those at highest risk of poor covid-19 outcomes who are most likely to benefit.^9^ ^10^ This strategy was based on a set of clinical conditions associated with a high relative risk of severe outcomes from the published literature,^6^ ^11^ combined with clinical judgment about the likelihood of clinical benefit based on the biological mechanism for neutralising monoclonal antibodies.^9^ Patients with these conditions were then identified from centrally held electronic health records and contacted by NHS Digital in December 2021 to inform them of their potential eligibility for neutralising monoclonal antibodies should they develop symptoms of SARS-CoV-2 infection. The guidance did not, however, account for the cumulative absolute risk associated with multiple comorbidities, age, previous infection, vaccination status, or the new variants of the SARS-CoV-2 virus.

This study was commissioned by the UK’s Department of Health and Social Care. The aims of the study were to develop and validate a new QCOVID risk algorithm (QCOVID4) based on new data from the period when the omicron variant of the SARS-CoV-2 virus was predominant in England, taking into account previous infection with the virus and number of doses of the covid-19 vaccine. We also evaluated the performance of the QCOVID4 algorithm with earlier versions of the risk model developed in the first two waves of the covid-19 pandemic, and with the high risk cohort identified by NHS Digital, based on the relative risks of a list of conditions. We hope that the results will be used to inform ongoing strategies for targeting treatments and other public health interventions, designed to protect those most at risk from covid-19 related death and hospital admission.

## Methods

### Data sources

We used the QResearch database (version 47) of 12 million current patients with personal, clinical, and drug use data. The database is used for epidemiological^1^ ^12^ and drug safety research.^13^ ^14^ QResearch is linked to multiple datasets at the individual patient level. For this analysis, we used the following linked datasets:

- National Immunisation Management System (NIMS) database of covid-19 vaccinations to identify data on dates and doses of vaccines for all people who received a vaccine in England
- Hospital Episode Statistics (HES) dataset supplemented by the more regularly updated Secondary Users Service (SUS-PLUS) data
- Civil registration national data for mortality, with date, and up to 15 causes of death
- SARS-CoV-2 infection data (Second Generation Surveillance System (SGSS) and Pillar 2)
- Systemic anticancer treatment data
- NHS Digital high risk cohort prioritised for new covid-19 treatments in December 2021.

### Study design and period for cohort

We undertook a cohort study of all individuals aged 18-100 years who had one or more positive SARS-CoV-2 test results from 11 December 2021 (the date of the first notified patient with the omicron variant of the SARS-CoV-2 virus in the UK) to 31 March 2022 (the date after which widespread free NHS tests for SARS-CoV-2 were not available). Individuals were followed from the date of their first SARS-CoV-2 test result during the study period to the outcome of interest, or to their death or the end of the study on 30 June 2022 (the latest date for which data on mortality and hospital admissions were available).

### Outcomes for cohort

The primary outcome was time to covid-19 related death (either in hospital or in the community), recorded in any position on the death certificate, or death within 28 days of a positive SARS-CoV-2 test result. The secondary outcome was time to hospital admission for covid-19, defined as confirmed or suspected covid-19 based on the ICD-10 (international classification of diseases, 10th edition) codes U071 and U072. We used these definitions of outcomes for consistency with other QCOVID algorithms and because they are used for covid-19 related death and hospital admission in the UK.^15^

### Predictor variables

Candidate predictor variables were those previously identified as associated with an increased risk of covid-19 related death or admission to hospital for covid-19 from the original QCOVID protocol^16^ and the published literature.^1^ ^6^ ^12^ ^17^ The variables were age, sex, ethnic group (based on categories in the 2021 census of England and Wales, www.ethnicity-facts-figures.service.gov.uk/style-guide/ethnic-groups), Townsend material deprivation score (an area level score based on postcode where higher scores indicate higher levels of deprivation^18^), number of vaccine doses (none, 1, 2, 3, or ≥4), previous SARS-CoV-2 infection, body mass index,^17^ residence (care home, homeless, or neither), chronic kidney disease, chemotherapy in the previous 12 months, type 1 or type 2 diabetes (with haemoglobin A_1C_ <59 or ≥59 mmol/mol), blood cancer, bone marrow transplant in the past six months, respiratory cancer, radiotherapy in the past six months, solid organ transplant, chronic obstructive pulmonary disease, asthma, rare lung diseases (cystic fibrosis, bronchiectasis, or alveolitis), pulmonary hypertension or pulmonary fibrosis, coronary heart disease, stroke, atrial fibrillation, heart failure, venous thromboembolism, peripheral vascular disease, congenital heart disease, dementia, Parkinson’s disease, epilepsy, Down’s syndrome, rare neurological conditions (motor neurone disease, multiple sclerosis, myasthenia, or Huntington’s chorea), cerebral palsy, osteoporotic fracture, rheumatoid arthritis or systemic lupus erythematosus, liver cirrhosis, bipolar disorder or schizophrenia, inflammatory bowel disease, sickle cell disease, HIV or AIDS, and severe combined immunodeficiency.

We defined predictors based on information recorded in primary care electronic health records at the start of follow-up (date of the first positive test result for SARS-CoV-2 during the study period), except for data for the number of SARS-CoV-2 infections, covid-19 vaccinations, chemotherapy, radiotherapy, and transplants, which were based on linked secondary care data. For all predictor variables, we used the most recently recorded available value at the cohort entry date.

### Model development

We used a random sample of 90% of practices to develop the models, and the remaining 10% of practices for validation of the model.^6^ We developed separate risk models for men and women, with Cox proportional hazard models to calculate hazard ratios for the two outcomes. Data for sex were taken from information in the QResearch database, which are likely to reflect the information reported by the patient to the general practice at registration. We decided not to use the landmarking approach or adjust for infection rate, as in QCOVID3,^6^ because our target population were those with a positive test result for SARS-CoV2 and follow-up was from the date of the positive test result. We used second degree fractional polynomials to model non-linear relations for continuous variables, including age, body mass index, and Townsend material deprivation score, by using the non-imputed complete data.^18^ We used multiple imputation with chained equations to impute missing values for ethnic group, Townsend score, body mass index, and haemoglobin A_1C_ level. We carried out five imputations and fitted the prediction models in each imputed dataset. We used Rubin’s rules^19^ to combine the model parameter estimates across the imputed datasets. 

We fitted full models and inspected the results. We then retained variables in the final models that were significant at the 5% level and where adjusted hazard ratios were >1.1 (for binary variables). We combined clinically similar variables with low numbers of events. We examined interactions between predictor variables and age. We assessed model optimism by calculating Van Houwelingen’s measure of heuristic shrinkage.^20^ We used post-estimation methods to estimate the baseline survivor function at 90 days from the Cox regression model, as described by Kalbfleisch and Prentice,^21^ based on zero values of centred continuous variables, with all binary predictor values set to zero. This value is analogous to the Kaplan-Meier product limit estimate.^21^ We used the mean of these values across all imputations in the calculation of predicted risks. Lastly, we combined regression coefficients from the final models with these estimates of the baseline survivor function evaluated at 90 days to derive risk equations for each outcome.

### Model evaluation

We evaluated the performance of the model in the validation cohort. We used multiple imputation to replace missing values for ethnic group, haemoglobin A_1C_ levels, body mass index, and Townsend score, with the same imputation model as in the derivation cohort. We applied the final risk equations to calculate the predicted risks for each outcome. We calculated Harrell’s C statistics,^22^ Royston’s R^2^ values, and associated D statistics,^23^ and combined them across imputed datasets with Rubin’s rules.^19^ We ran a Cox proportional hazards model to calculate the calibration slope with the prognostic index over the study period. We generated smoothed calibration plots with the running command in Stata to compare observed event probabilities and predicted risks at 90 days across the range of predicted risks. Pseudovalues were used to generate the observed event probabilities determined at 90 days with the stpsurv command in Stata.^24^

We calculated each performance metric in the whole validation cohort and in subgroups for age, ethnic group (where numbers allowed), and geographical region. We compared model discrimination with predicted risks calculated with earlier versions of QCOVID developed on populations that were not vaccinated: during the first wave of the pandemic, QCOVID1 was developed on the total population that was not vaccinated, between 24 January 2020 and 30 April 2020; and during the second pandemic wave, QCOVID2 was developed on patients with a positive test result for SARS-CoV-2 who were not vaccinated, between 8 December 2020 and 21 June 2021, when the alpha and delta variants of the SARS-CoV-2 virus were dominant. The predicted risks based on these models did not therefore account for the vaccination status of individuals in the validation cohort.

We decided not to include QCOVID3 because it was modelled with a population who were vaccinated. Also, QCOVID3 was designed to follow up participants from 14 days after the date of vaccination whereas the follow-up for QCOVID4 was from the date of a positive SARS-CoV-2 test result. QCOVID4 also included people who were not vaccinated as well as people who had received up to four doses of vaccine, so we considered that QCOVID3 was less applicable to our study population.

### Risk stratification

We applied the QCOVID4 algorithms to the validation cohort to define the centile thresholds based on absolute predicted risk. We calculated sensitivity as the total number of patients with a predicted risk above the risk threshold with a covid-19 related death out of the total number of covid-19 related deaths.

We also compared risk stratification using QCOVID4 to identify the top 2.5% of patients at highest absolute predicted risk in the validation cohort (because this percentage reflected the actual percentage of high risk patients identified by NHS Digital) with the current recommended guidelines which have selected a high risk cohort based on relative risks of selected medical conditions.

### Decision curve analysis

We used decision curve analysis in the validation cohort accounting for censoring to evaluate the net benefits of the new risk equations.^25^ This approach assesses the benefits of correctly detecting people who will have a covid-19 related death (for example) compared with the harms from a false positive classification (which could lead to unnecessary intervention). The net benefit of a risk equation at a specific risk threshold is calculated from the difference between the proportion of true positive results and the proportion of false positive results multiplied by the odds defined by the risk threshold value.^26^ We calculated the net benefits of QCOVID4 compared with QCOVID2 in the whole cohort and in regional subgroups, across a range of threshold probabilities, and compared these with alternative strategies, such as assuming that all patients are treated or that no patients are treated. In general, the strategy with the highest net benefit at any given risk threshold is considered to have the most clinical value.

### Reporting

We adhered to the RECORD (REporting of studies Conducted using Observational Routinely collected health Data)^27^ and TRIPOD (Transparent Reporting of a multivariable prediction model for Individual Prognosis or Diagnosis) statements for reporting.^28^ We used all of the available data on the database to maximise the power and generalisability of the results. We used Stata (version 17) for analyses.

### Patient and public involvement

Patients were involved in framing the research question, identifying predictors, and in developing plans for design and implementation of the QCOVID risk tool. A citizen’s jury convened by the Scottish government evaluated earlier versions of the QCOVID algorithm and highlighted the importance of keeping it up-to-date and maintaining transparency over its use.^29^

## Results

### Baseline characteristics of study cohorts

The QResearch database (version 47) included 1430 practices. We allocated 1287 practices to the derivation cohort and 143 to the validation cohort. Of the 9 526 580 patients aged 18-100 years in the derivation cohort, 1 297 922 (13.6%) had a positive test result for SARS-CoV-2 during the study period. Of these, 18 756 (1.5%) were admitted to hospital for covid-19 and 3878 (0.3%) had a covid-19 related death during follow-up. Of the 1 064 255 patients in the validation cohort, 145 397 (13.7%) had a positive test result for SARS-CoV-2 and were included in the analysis. Of these, 2124 (1.5%) were admitted to hospital for covid-19 and 461 (0.3%) had a covid-19 related death.

Table 1 shows the baseline characteristics of those who had a positive test result for SARS-CoV-2, those with a covid-19 related death, and those admitted to hospital for covid-19 in the derivation cohort. Supplementary table 1 shows the corresponding results for the validation cohort. Mean age in the derivation cohort for those with a positive SARS-CoV-2 test result was 42.4 years (standard deviation 16.4), for those admitted to hospital for covid-19, 55.6 years (22.1), and for those with a covid-19 related death, 80.9 years (12.3).

**Table 1:** Baseline characteristics of derivation cohort of patients with positive test result for SARS-CoV-2, covid-19 related death, and hospital admission for covid-19

Characteristic	Total population	SARS-CoV-2 positive test result	Covid-19 related death	Hospital admission for covid-19
Total No of patients	9 526 580	1 297 922	3878	18 756
Men	4 776 225 (50.14)	555 918 (42.83)	2026 (52.24)	7708 (41.10)
Mean (SD) age (years)	47.22 (18.57)	42.39 (16.44)	80.93 (12.29)	55.63 (22.13)
Ethnic group:				
White	6 071 398 (63.73)	883 218 (68.05)	2944 (75.92)	12913 (68.85)
Indian	302 042 (3.17)	35 493 (2.73)	49 (1.26)	411 (2.19)
Pakistani	183 637 (1.93)	16 780 (1.29)	40 (1.03)	405 (2.16)
Bangladeshi	120 383 (1.26)	11 411 (0.88)	28 (0.72)	253 (1.35)
Other Asian	194 823 (2.05)	23 926 (1.84)	34 (0.88)	336 (1.79)
Black Caribbean	104 802 (1.10)	12 256 (0.94)	64 (1.65)	378 (2.02)
Black African	250 975 (2.63)	25 970 (2.00)	36 (0.93)	531 (2.83)
Chinese	110 390 (1.16)	8824 (0.68)	9 (0.23)	70 (0.37)
Other ethnic group	403 014 (4.23)	51 419 (3.96)	47 (1.21)	759 (4.05)
Ethnic group not recorded	1 785 116 (18.74)	228 625 (17.61)	627 (16.17)	2700 (14.40)
Townsend material deprivation score (five groups):				
1 (most affluent)	2 241 175 (23.53)	313 128 (24.13)	987 (25.45)	3827 (20.40)
2	2 030 972 (21.32)	289 819 (22.33)	888 (22.90)	3764 (20.07)
3	1 850 166 (19.42)	260 461 (20.07)	785 (20.24)	3863 (20.60)
4	1 682 062 (17.66)	220 973 (17.03)	689 (17.77)	3715 (19.81)
5 (most deprived)	1 603 423 (16.83)	192 601 (14.84)	506 (13.05)	3358 (17.90)
6 (not recorded)	118 782 (1.25)	20 940 (1.61)	23 (0.59)	229 (1.22)
Residence:				
Not care home or homeless	9 436 601 (99.06)	1 279 698 (98.60)	2997 (77.28)	17803 (94.92)
Care home	70424 (0.74)	16703 (1.29)	876 (22.59)	873 (4.65)
Homeless	19555 (0.21)	1521 (0.12)	5 (0.13)	80 (0.43)
Body mass index:				
<18.5	239 052 (2.51)	32 788 (2.53)	287 (7.40)	570 (3.04)
18.5-24.99	2 770 605 (29.08)	398 814 (30.73)	1251 (32.26)	5083 (27.10)
25-29.99	2 339 194 (24.55)	31 1541 (24.00)	942 (24.29)	4736 (25.25)
30-34.99	1 096 337 (11.51)	146 966 (11.32)	470 (12.12)	2682 (14.30)
35-39.99	425 986 (4.47)	60 829 (4.69)	156 (4.02)	1223 (6.52)
³40	224 232 (2.35)	33 922 (2.61)	82 (2.11)	729 (3.89)
Not recorded	2 431 174 (25.52)	313 062 (24.12)	690 (17.79)	3733 (19.90)
Chronic kidney disease:				
No chronic kidney disease	9 166 042 (96.22)	1 267 768 (97.68)	2557 (65.94)	16 082 (85.74)
Chronic kidney disease stage 5 only	10 386 (0.11)	1384 (0.11)	67 (1.73)	238 (1.27)
Chronic kidney disease stage 5 with dialysis	3178 (0.03)	484 (0.04)	10 (0.26)	49 (0.26)
Chronic kidney disease stage 5 with transplantation	5391 (0.06)	1123 (0.09)	26 (0.67)	251 (1.34)
Learning disability:				
No	9 353 294 (98.18)	1 275 513 (98.27)	3773 (97.29)	18 189 (96.98)
Yes	169 009 (1.77)	21 745 (1.68)	102 (2.63)	529 (2.82)
Down’s syndrome	4277 (0.04)	664 (0.05)	*	38 (0.20)
Chemotherapy in past 12 months:				
None	9 490 847 (99.62)	1 293 639 (99.67)	3633 (93.68)	18 205 (97.06)
Grade A	16 515 (0.17)	1683 (0.13)	76 (1.96)	169 (0.90)
Grade B	18 150 (0.19)	2445 (0.19)	155 (4.00)	356 (1.90)
Grade C	1068 (0.01)	155 (0.01)	14 (0.36)	26 (0.14)
Diabetes status:				
No type 1 diabetes	9 480 133 (99.51)	1 290 601 (99.44)	3855 (99.41)	18 491 (98.59)
Type 1 HbA £59 mmol/mol	13 894 (0.15)	2354 (0.18)	5 (0.13)	83 (0.44)
Type 1 HbA_1C_ >59 mmol/mol	27 110 (0.28)	4136 (0.32)	14 (0.36)	149 (0.79)
Type 1 HbA_1C_ not recorded	5443 (0.06)	831 (0.06)	*	33 (0.18)
No type 2 diabetes	8 901 793 (93.44)	1 241 431 (95.65)	2814 (72.56)	15 849 (84.50)
Type 2 HbA £59 mmol/mol	349 984 (3.67)	31 490 (2.43)	639 (16.48)	1578 (8.41)
Type 2 HbA_1C_ >59 mmol/mol	202 963 (2.13)	18 246 (1.41)	288 (7.43)	929 (4.95)
Type 2 HbA_1C_ not recorded	71 840 (0.75)	6755 (0.52)	137 (3.53)	400 (2.13)
No of covid-19 vaccine doses:				
0	1 719 164 (18.05)	145 887 (11.24)	413 (10.65)	3720 (19.83)
1	279 909 (2.94)	44 125 (3.40)	110 (2.84)	803 (4.28)
2	1 519 472 (15.95)	363 949 (28.04)	670 (17.28)	3832 (20.43)
3	5 194 720 (54.53)	737 212 (56.80)	2617 (67.48)	9861 (52.58)
≥4	813 315 (8.54)	6749 (0.52)	68 (1.75)	540 (2.88)
SARS-CoV-2 infection before study entry	1 322 848 (13.89)	145 587 (11.22)	177 (4.56)	1384 (7.38)
Blood cancer	72 167 (0.76)	8389 (0.65)	261 (6.73)	741 (3.95)
Bone marrow transplantation in past six months	332 (0.00)	44 (0.00)	*	11 (0.06)
Respiratory cancer	20 163 (0.21)	1743 (0.13)	101 (2.60)	167 (0.89)
Radiotherapy in past six months	11 890 (0.12)	1441 (0.11)	81 (2.09)	144 (0.77)
Solid organ transplantation	2104 (0.02)	358 (0.03)	10 (0.26)	62 (0.33)
Chronic obstructive pulmonary disease	209 519 (2.20)	17 070 (1.32)	683 (17.61)	1529 (8.15)
Asthma	1 292 225 (13.56)	210 812 (16.24)	567 (14.62)	3655 (19.49)
Rare pulmonary conditions	51 203 (0.54)	5374 (0.41)	139 (3.58)	364 (1.94)
Pulmonary hypertension	8356 (0.09)	753 (0.06)	36 (0.93)	83 (0.44)
Coronary heart disease	325 875 (3.42)	28 443 (2.19)	935 (24.11)	1962 (10.46)
Stroke	201 933 (2.12)	18 250 (1.41)	703 (18.13)	1432 (7.63)
Atrial fibrillation	228 054 (2.39)	20 448 (1.58)	879 (22.67)	1551 (8.27)
Congestive cardiac failure	119 691 (1.26)	10 588 (0.82)	628 (16.19)	1096 (5.84)
Venous thromboembolism	183 548 (1.93)	20 313 (1.57)	434 (11.19)	1150 (6.13)
Peripheral vascular disease	65 488 (0.69)	4885 (0.38)	243 (6.27)	461 (2.46)
Congenital heart disease	44 255 (0.46)	7137 (0.55)	19 (0.49)	146 (0.78)
Dementia	99 967 (1.05)	15 272 (1.18)	1114 (28.73)	1400 (7.46)
Parkinson’s disease	22 804 (0.24)	2248 (0.17)	140 (3.61)	185 (0.99)
Epilepsy	124 836 (1.31)	16 878 (1.30)	116 (2.99)	537 (2.86)
Rare neurological conditions	28 958 (0.30)	4074 (0.31)	35 (0.90)	402 (2.14)
Cerebral palsy	10 915 (0.11)	1438 (0.11)	*	43 (0.23)
Osteoporotic fracture	373 642 (3.92)	47 758 (3.68)	695 (17.92)	1429 (7.62)
Rheumatoid arthritis or systemic lupus erythematosus	238 262 (2.50)	27 153 (2.09)	335 (8.64)	1206 (6.43)
Cirrhosis	21 588 (0.23)	2145 (0.17)	68 (1.75)	226 (1.20)
Bipolar disorder or schizophrenia	108 993 (1.14)	12 527 (0.97)	75 (1.93)	427 (2.28)
Inflammatory bowel disease	90 015 (0.94)	13 643 (1.05)	70 (1.81)	519 (2.77)
Sickle cell disease, HIV, or severe combined immunodeficiency	26 579 (0.28)	3760 (0.29)	23 (0.59)	166 (0.89)

Data are number (%) of participants unless stated otherwise. SD=standard deviation; HbA=haemoglobin A; HbA_1C_=haemoglobin A_1C_.

Ethnic groups based on categories in the 2021 census of England and Wales (www.ethnicity-facts-figures.service.gov.uk/style-guide/ethnic-groups).

* Cells with counts <5 suppressed.

### Factors associated with increased or decreased risk of severe covid-19 outcomes

**Fig 1:**
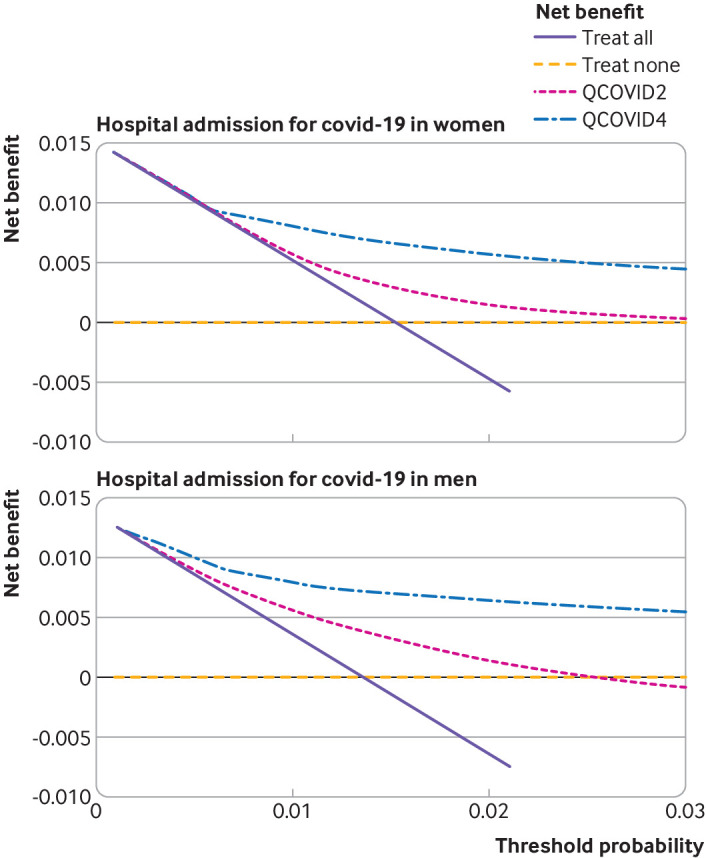
Adjusted hazard ratios for predictor variables in the final QCOVID4 model for covid-19 related death in women. Ethnic groups based on categories in the 2021 census of England and Wales (www.ethnicity-facts-figures.service.gov.uk/style-guide/ethnic-groups). CI=confidence interval

**Fig 2:**
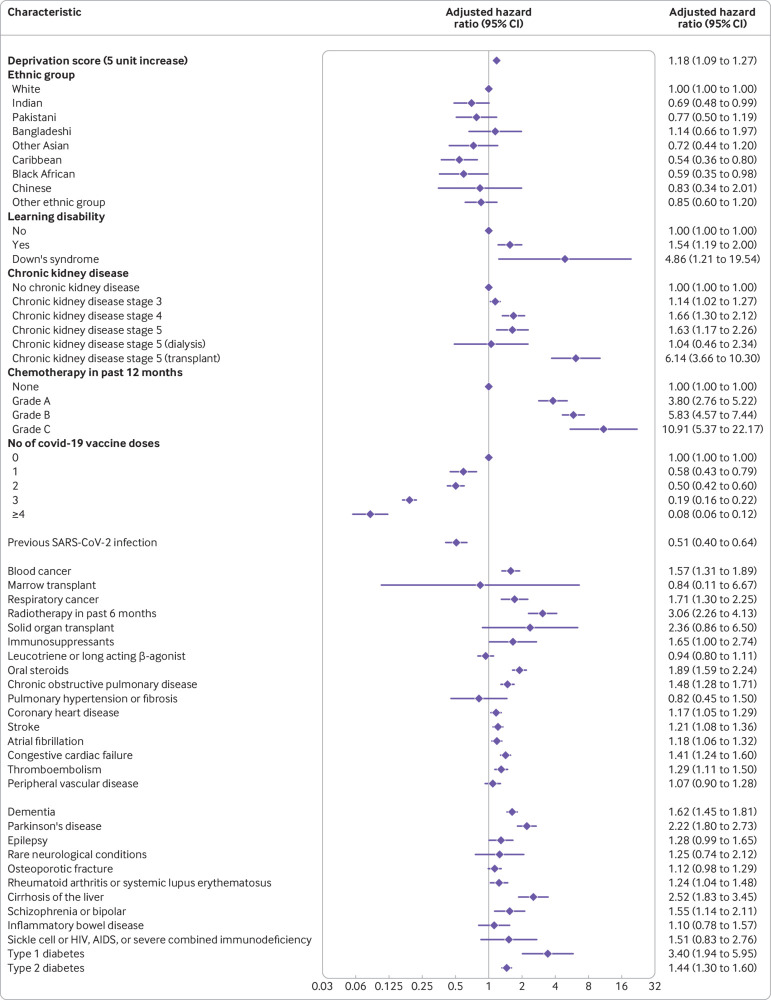
Adjusted hazard ratios for predictor variables in the final QCOVID4 model for covid-19 related death in men. Ethnic groups based on categories in the 2021 census of England and Wales (www.ethnicity-facts-figures.service.gov.uk/style-guide/ethnic-groups). CI=confidence interval

**Fig 3:**
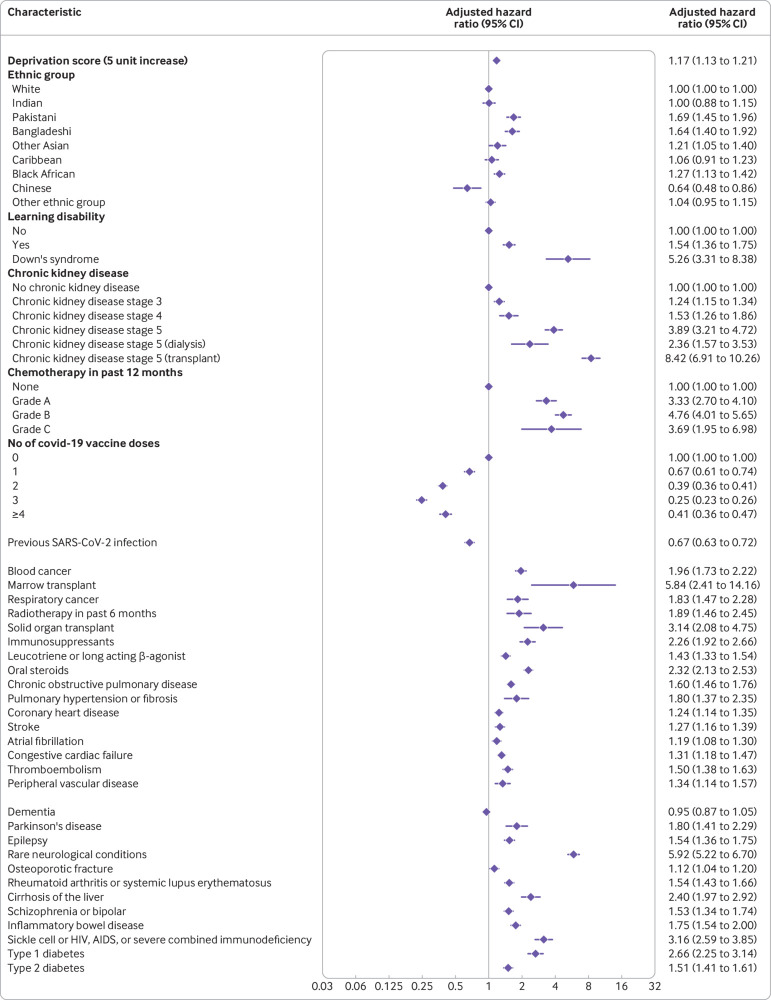
Adjusted hazard ratios for predictor variables in the final QCOVID4 model for hospital admission for covid-19 in women. Ethnic groups based on categories in the 2021 census of England and Wales (www.ethnicity-facts-figures.service.gov.uk/style-guide/ethnic-groups). CI=confidence interval

**Fig 4:**
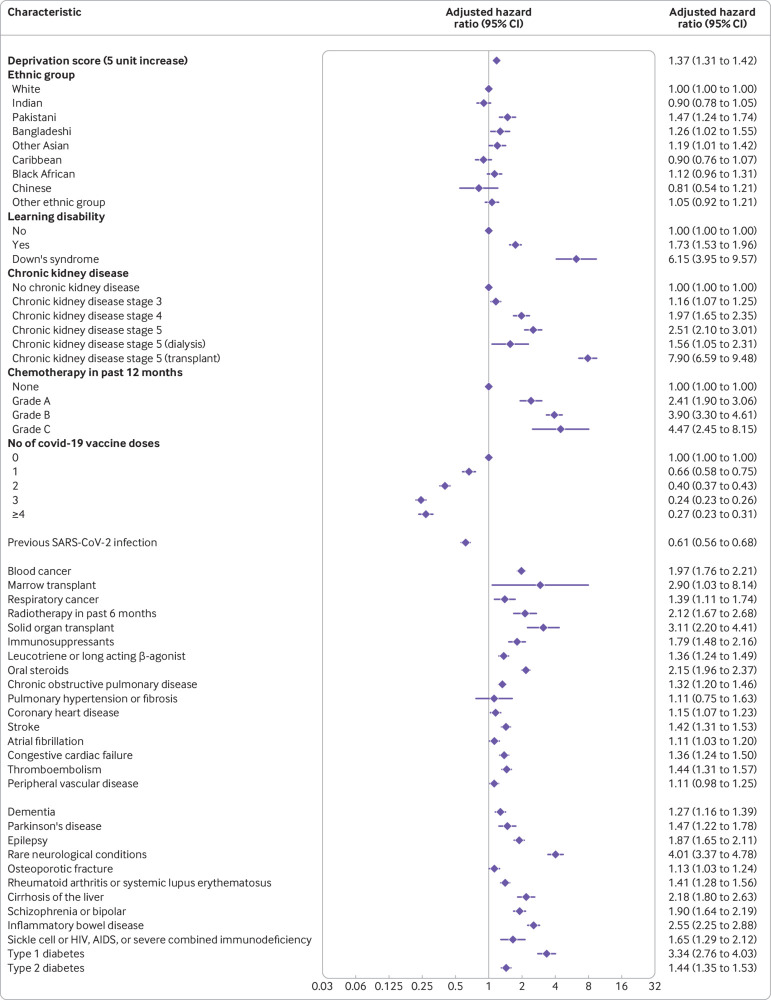
Adjusted hazard ratios for predictor variables in the final QCOVID4 model for hospital admission for covid-19 in men. Ethnic groups based on categories in the 2021 census of England and Wales (www.ethnicity-facts-figures.service.gov.uk/style-guide/ethnic-groups). CI=confidence interval

Figure 1 shows the adjusted hazard ratios for variables in the final QCOVID4 model for covid-19 related death in women. Figure 2 show the corresponding results for men. Figure 3 and figure 4 show the adjusted hazard ratios for variables in the final model for hospital admission for covid-19 in women and men, respectively. Supplementary figure 1 shows the adjusted hazard ratios for fractional polynomial terms for each of the models for age and body mass index. The full model, model coefficients, functional form, and baseline survival function are published at https://bmj2022.qcovid.org. Values for Van Houwelingen’s heuristic shrinkage^20^ were close to one (for covid-19 related death the value was 0.99 in women and men; the corresponding values for hospital admission for covid-19 were both 1.00). Based on our large sample size and these shrinkage values, we considered that modifying the final model to account for model optimism was not needed.

The rate of covid-19 related death in men increased steeply with age and was associated with deprivation score. In the final model in men, adjusted hazard ratios were highest for those with these conditions (fig 2 shows the 95% confidence intervals): kidney transplant (6.1-fold increase), Down’s syndrome (4.9-fold), radiotherapy (3.1-fold), type 1 diabetes (3.4-fold), chemotherapy grade A (3.8-fold), chemotherapy grade B (5.8-fold), chemotherapy grade C (10.9-fold), solid organ transplant ever (2.4-fold), dementia (1.6-fold), Parkinson’s disease (2.2-fold), and cirrhosis of the liver (2.5-fold). Other conditions associated with increased covid-19 related death in men included learning disability, chronic kidney disease (stages 4 and 5), blood cancer, respiratory cancer, immunosuppressants, oral steroids, chronic obstructive pulmonary disease, coronary heart disease, stroke, atrial fibrillation, heart failure, thromboembolism, rheumatoid or systemic lupus erythematosus, schizophrenia or bipolar disease, sickle cell, HIV, or severe combined immunodeficiency, and type 2 diabetes. We found no association between covid-19 related death and residence, asthma, rare pulmonary conditions, cerebral palsy, or congenital heart disease. We found no difference in risk according to haemoglobin A_1C_ levels, so we included type 1 and type 2 diabetes as binary rather than categorical variables. These results were generally similar in women (fig 1).

We found no significantly increased risks of covid-19 related death in men or women in other ethnic groups compared with the white group. An increased risk of hospital admission for covid-19 was found among Bangladeshi, Pakistani, and other Asian men and women, and for black African women (fig 3 and fig 4).

The rate of covid-19 related death was lower among those who were vaccinated against covid-19 than those who were not vaccinated, with evidence of a dose-response relation (supplementary table 2). For example, compared with men who were not vaccinated, we found a 42% risk reduction associated with one dose of vaccine (adjusted hazard ratio 0.58, 95% confidence interval 0.43 to 0.79) and a 92% reduction in risk with four or more doses (0.08, 0.06 to 0.12). The reduced risks of covid-19 related death associated with covid-19 vaccination doses were similar in women. The risk of hospital admission for covid-19 was also reduced in men and women who were vaccinated.

Previous SARS-CoV-2 infection was associated with a 49% reduced risk of covid-19 related death in men (adjusted hazard ratio 0.51, 95% confidence interval 0.40 to 0.64) and a 45% reduced risk in women (0.55, 0.45 to 0.67), independent of age, ethnic group, vaccination status, and other factors included in the final QCOVID4 models. The 39% reduction in the risk of hospital admission associated with previous SARS-CoV-2 infection for men (0.61, 0.56 to 0.68) was similar to that in women (33%; 0.67, 0.63 to 0.72).

### Discrimination

Table 2 shows the explained variation and discrimination of QCOVID1, QCOVID2, and QCOVID4 models in the validation cohort for women and men overall for covid-19 related death and hospital admission. The QCOVID4 algorithm explained 76.6% (95% confidence interval 74.4% to 78.8%) of the variation (R^2^) in time to covid-19 related death in women. The D statistic was 3.70 (3.48 to 3.93) and Harrell’s C statistic was 0.965 (0.951 to 0.978). The corresponding results for covid-19 related death in men were similar, with R^2^ of 76.0% (73.9% to 78.2%), D statistic 3.65 (3.43 to 3.86), and C statistic 0.970 (0.962 to 0.979).

**Table 2:** Performance of the QCOVID1, QCOVID2, and QCOVID4 algorithms in men and women in validation cohort

Statistic	Covid-19 related deaths			Hospital admission for covid-19	
	Women	Men		Women	Men
**QCOVID1**					
R^2^ (%)	73.6 (71.0 to 76.1)	73.0 (70.6 to 75.4)		27.6 (24.7 to 30.5)	48.4 (45.6 to 51.2)
D statistic	3.41 (3.19 to 3.64)	3.36 (3.16 to 3.57)		1.26 (1.17 to 1.36)	1.98 (1.87 to 2.09)
Harrell’s C	0.945 (0.928 to 0.963)	0.948 (0.933 to 0.963)		0.684 (0.669 to 0.7)	0.798 (0.781 to 0.814)
Calibration slope*	NA	NA		NA	NA
**QCOVID2**					
R^2^ (%)	75.4 (73.1 to 77.7)	74.1 (71.8 to 76.4)		67.4 (64.1 to 70.7)	68.2 (65.2 to 71.2)
D statistic	3.58 (3.36 to 3.81)	3.46 (3.25 to 3.67)		2.94 (2.72 to 3.16)	3.00 (2.79 to 3.21)
Harrell’s C	0.968 (0.959 to 0.978)	0.959 (0.946 to 0.971)		0.939 (0.930 to 0.948)	0.932 (0.918 to 0.945)
Calibration slope	NA	NA		NA	NA
**QCOVID4**					
R^2^ (%)	76.6 (74.4 to 78.8)	76.0 (73.9 to 78.2)		73.9 (71.5 to 76.3)	74.4 (72.1 to 76.6)
D statistic	3.70 (3.48 to 3.93)	3.65 (3.43 to 3.86)		3.44 (3.23 to 3.66)	3.49 (3.28 to 3.69)
Harrell’s C	0.965 (0.951 to 0.978)	0.970 (0.962 to 0.979)		0.965 (0.956 to 0.973)	0.970 (0.963 to 0.977)
Calibration slope	1.01 (0.93 to 1.09)	1.00 (0.936 to 1.07)		0.996 (0.956 to 1.04)	1.01 (0.972 to 1.06)

Values are mean (95% confidence interval). NA=not applicable.

* Calibration not assessed because QCOVID1 and QCOVID2 were based on the whole population rather than restricted to the subset with a positive test result for SARS-CoV-2 infection.

The performance of QCOVID4 for covid-19 related death was slightly improved compared with both QCOVID1 and QCOVID2. For hospital admissions for covid-19, however, QCOVID4 had substantially improved performance compared with QCOVID2, which had improved performance compared with QCOVID1. For example, Harrell’s C statistic in men for QCOVID4 was 0.970 (95% confidence interval 0.963 to 0.977) compared with 0.932 (0.918 to 0.945) for QCOVID2 and 0.798 (0.781 to 0.814), for QCOVID1.

Supplementary table 3 shows the performance of QCOVID4 in subgroups by age and ethnic group. Supplementary table 4 shows the performance of QCOVID4 in subgroups by geographical region. Performance measures were generally higher in younger age groups and similar across ethnic and regional groups (where sufficient numbers in the subgroup existed to conduct an analysis).

### Calibration

**Fig 5:**
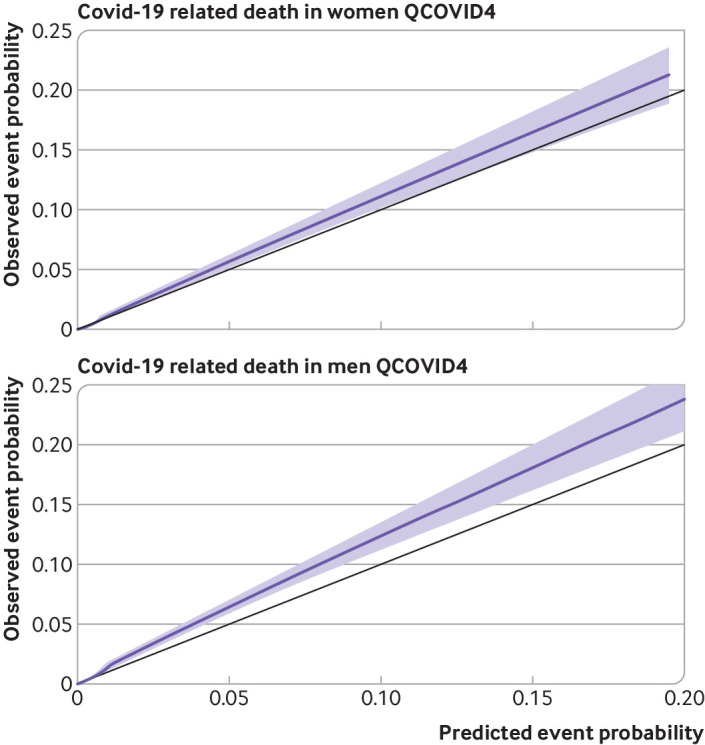
Smoothed calibration plots for covid-19 related death with QCOVID4 in women and men to assess calibration in validation cohort

**Fig 6:**
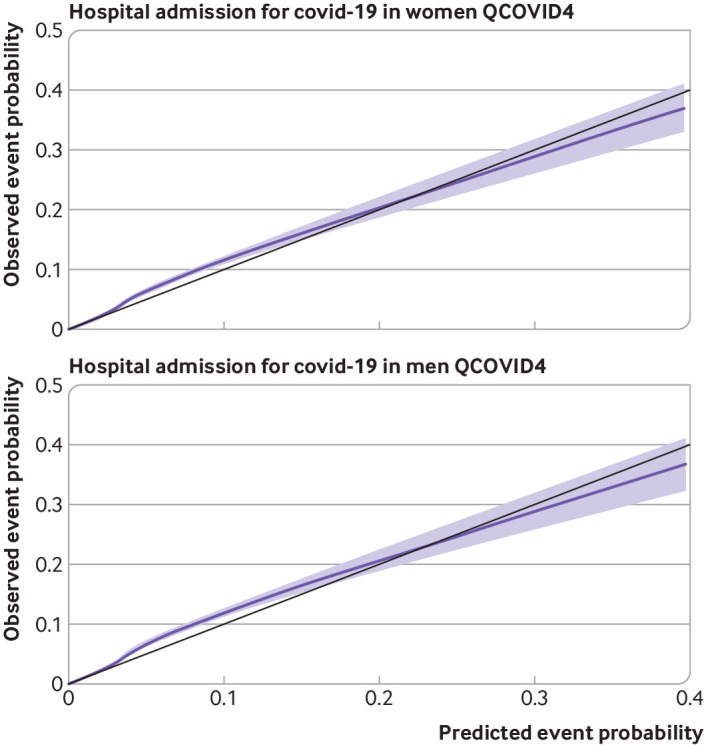
Smoothed calibration plots for hospital admission for covid-19 with QCOVID4 in women and men to assess calibration in validation cohort

Figure 5 and figure 6 show the smoothed calibration plots for covid-19 related death and hospital admission for covid-19 with QCOVID4 to assess calibration in the validation cohort. The results showed that the QCOVID4 model was well calibrated to the current contemporaneous validation dataset overall, with a small degree of underprediction for covid-19 related death, and underprediction at lower risks and overprediction at higher risks for hospital admission for covid-19.

**Fig 7:**
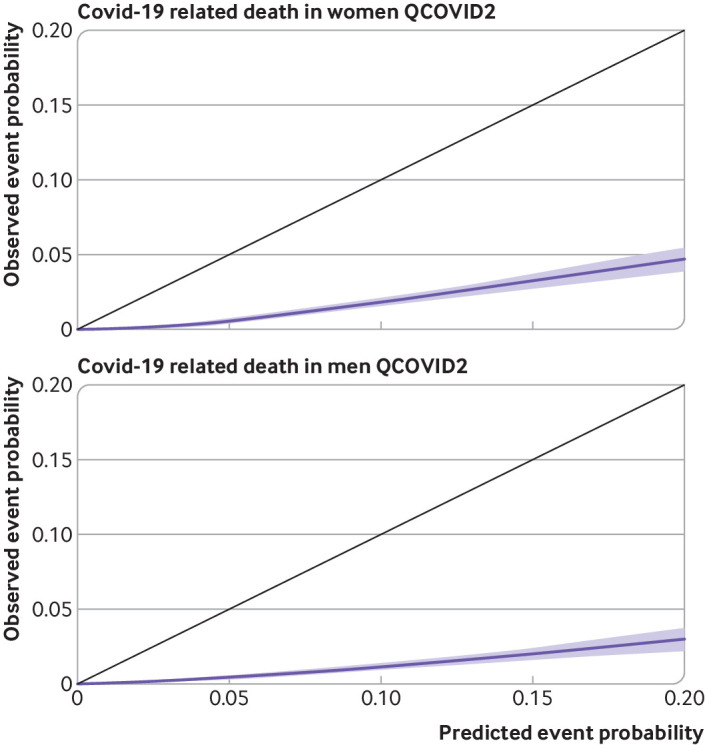
Smoothed calibration plots for covid-19 related death with QCOVID2 in women and men to assess calibration in validation cohort

**Fig 8:**
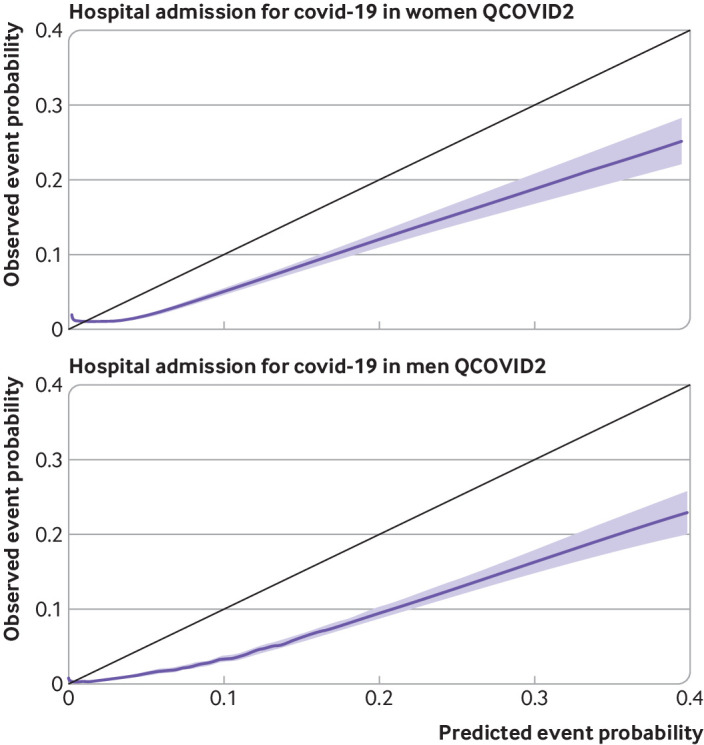
Smoothed calibration plots for hospital admission for covid-19 with QCOVID2 in women and men to assess calibration in validation cohort

Figure 7 and figure 8 show the corresponding results for QCOVID2 which was less well calibrated with substantial overprediction of risks for both covid-19 related death and admission to hospital. Supplementary figure 2 shows the corresponding results for QCOVID4 by ethnic group in men and women and a histogram of the distribution of predicted risks. Supplementary figure 3 shows the corresponding results for QCOVID4 by region in men and women. Supplementary figure 4 shows the corresponding results for QCOVID4 by age group. We found evidence of miscalibration in some subgroups, although low numbers of events in some ethnic groups and regions existed, resulting in wide confidence intervals.

### Thresholds

Table 3 shows the classification statistics in the validation cohort for men and women for 5% increments in centile values for the predicted covid-19 mortality risk with QCOVID4. For example, for the 20% of the cohort at the highest predicted risk (ie, those with a 90 day predicted risk of 0.075% or higher), sensitivity was 97.8%, specificity was 80.2%, and the observed risk at 90 days was 1.54%. The corresponding values for the top 5% at the highest predicted risk were sensitivity 87.6%, specificity 95.3%, and observed 90 day risk of 5.52%.

**Table 3:** Sensitivity, specificity, and observed 90 day covid-19 mortality risk at different centiles of predicted risk with QCOVID4 to predict covid-19 related death (n=461) in validation cohort of 145 397 people with a positive test result for SARS-CoV-2 infection

Top centile	Predicted 90 day risk threshold (%)	Sensitivity	Specificity	Observed risk (95% CI) at 90 days (%)
Top 5%	0.908	87.6	95.3	5.520 (5.018 to 6.070)
Top 10%	0.278	94.8	90.3	2.986 (2.722 to 3.276)
Top 15%	0.129	96.7	85.3	2.032 (1.853 to 2.228)
Top 20%	0.075	97.8	80.2	1.541 (1.406 to 1.689)
Top 25%	0.049	98.5	75.2	1.241 (1.132 to 1.360)
Top 30%	0.034	98.5	70.2	1.034 (0.943 to 1.133)
Top 35%	0.025	98.5	65.2	0.886 (0.809 to 0.972)
Top 40%	0.019	98.7	60.2	0.777 (0.709 to 0.852)
Top 45%	0.015	99.1	55.2	0.694 (0.633 to 0.761)
Top 50%	0.012	99.3	50.2	0.626 (0.571 to 0.686)
Top 55%	0.010	99.3	45.1	0.569 (0.519 to 0.624)
Top 60%	0.008	99.6	40.1	0.523 (0.477 to 0.573)
Top 65%	0.007	99.6	35.1	0.483 (0.440 to 0.529)
Top 70%	0.006	99.6	30.1	0.448 (0.409 to 0.491)
Top 75%	0.005	99.8	25.1	0.419 (0.382 to 0.459)
Top 80%	0.004	100.0	20.1	0.394 (0.359 to 0.431)
Top 85%	0.003	100.0	15.0	0.371 (0.338 to 0.406)
Top 90%	0.0025	100.0	10.0	0.350 (0.319 to 0.384)
Top 95%	0.0017	100.0	5.0	0.332 (0.303 to 0.363)

CI=confidence interval.

We identified 34 864 patients in the NHS Digital high risk cohort in the QResearch database, of whom 3600 were in the validation cohort. Table 4 shows the characteristics of these 3600 patients compared with the characteristics of 3600 patients (top 2.48%) with the highest predicted risks of covid-19 related death with the QCOVID4 models. Patients in the QCOVID4 high risk group tended to be much older (mean 85.0 *v* 55.4 years) with higher levels of comorbidities, with some exceptions (eg, chronic kidney disease stage 5, blood cancer, grade B chemotherapy, and rare neurological conditions). In total, 520 patients were included in both the QCOVID4 and NHS Digital high risk groups. Of the 461 covid-19 related deaths in the validation cohort, 333 (72.2%) were in the QCOVID4 high risk group, 307 (66.6%) in the QCOVID2 high risk group (top 2.48%), and 95 (20.6%) in the NHS Digital high risk group. Uptake of covid-19 treatments (both antiviral agents and neutralising monoclonal antibodies) was low, with 504 (14.0%) patients in the NHS Digital high risk group receiving treatment and 131 (3.6%) in the QCOVID4 high risk cohort.

**Table 4:** Characteristics of 3600 patients in NHS Digital high risk cohort and 3600 patients with highest predicted risks of covid-19 related death with QCOVID4 in validation cohort

Characteristic	Validation cohort	High risk QCOVID4	High risk NHS Digital
Total No of patients	145 397	3600	3600
Men	62 305 (42.85)	1682 (46.72)	1500 (41.67)
Covid-19 related deaths	461 (0.32)	333 (9.25)	95 (2.64)
Hospital admissions for covid-19	2873 (1.98)	815 (22.64)	462 (12.83)
Mean (SD) age (years)	42.96 (16.41)	85.00 (7.30)	55.40 (17.87)
Mean (SD) Townsend material deprivation score	−0.01 (3.03)	−0.15 (2.94)	−0.05 (3.04)
Mean (SD) body mass index	26.62 (5.67)	25.69 (5.87)	27.86 (6.06)
Age (years):			
20-29	34 453 (23.70)	—	286 (7.94)
30-39	35 586 (24.48)	—	495 (13.75)
40-49	28 922 (19.89)	—	596 (16.56)
50-59	22 507 (15.48)	11 (0.31)	692 (19.22)
60-69	12 745 (8.77)	97 (2.69)	663 (18.42)
70-79	7056 (4.85)	653 (18.14)	523 (14.53)
80-100	4128 (2.84)	2839 (78.86)	345 (9.58)
Ethnic group:			
White	123 145 (84.70)	3341 (92.81)	3006 (83.50)
Indian	3523 (2.42)	54 (1.50)	59 (1.64)
Pakistani	2831 (1.95)	48 (1.33)	78 (2.17)
Bangladeshi	1894 (1.30)	26 (0.72)	47 (1.31)
Other Asian	2476 (1.70)	19 (0.53)	36 (1.00)
Black Caribbean	1142 (0.79)	40 (1.11)	52 (1.44)
Black African	3256 (2.24)	35 (0.97)	160 (4.44)
Chinese	1128 (0.78)	11 (0.31)	32 (0.89)
Other ethnic group	6002 (4.13)	26 (0.72)	130 (3.61)
Chronic kidney disease:			
No chronic kidney disease	141 747 (97.49)	2244 (62.33)	3017 (83.81)
Chronic kidney disease stage 3	3153 (2.17)	1179 (32.75)	282 (7.83)
Chronic kidney disease stage 4	167 (0.11)	96 (2.67)	37 (1.03)
Chronic kidney disease stage 5 only	164 (0.11)	59 (1.64)	110 (3.06)
Chronic kidney disease stage 5 with dialysis	53 (0.04)	5 (0.14)	41 (1.14)
Chronic kidney disease stage 5 with transplantation	113 (0.08)	17 (0.47)	113 (3.14)
Learning disability:			
No	142 758 (98.18)	3496 (97.11)	3443 (95.64)
Yes	2553 (1.76)	102 (2.83)	72 (2.00)
Down’s syndrome	86 (0.06)	*	85 (2.36)
Chemotherapy in past 12 months:			
None	144 888 (99.65)	3435 (95.42)	3342 (92.83)
Grade A	207 (0.14)	53 (1.47)	31 (0.86)
Grade B	284 (0.20)	106 (2.94)	212 (5.89)
Grade C	18 (0.01)	6 (0.17)	15 (0.42)
No of covid vaccine doses:			
0	15 725 (10.82)	211 (5.86)	161 (4.47)
1	4985 (3.43)	82 (2.28)	67 (1.86)
2	39 637 (27.26)	513 (14.25)	485 (13.47)
3	84 272 (57.96)	2735 (75.97)	2567 (71.31)
≥4	778 (0.54)	59 (1.64)	320 (8.89)
No covid-19 drug treatments in follow-up Covid-19 drug treatments in follow-up:	144 564 (99.43)	3469 (96.36)	3096 (86.00)
Tocilizumab	54 (0.04)	13 (0.36)	14 (0.39)
Sotrovimab	406 (0.28)	50 (1.39)	292 (8.11)
Sarilumab	22 (0.02)	7 (0.19)	*
Casirivimab and Imdevimab	20 (0.01)	10 (0.28)	8 (0.22)
Remdesivir	108 (0.07)	29 (0.81)	24 (0.67)
Molnupiravir	110 (0.08)	13 (0.36)	95 (2.64)
Paxlovid	113 (0.08)	9 (0.25)	68 (1.89)
SARS-CoV-2 infection before study entry	16 014 (11.01)	199 (5.53)	333 (9.25)
Blood cancer	998 (0.69)	216 (6.00)	526 (14.61)
Bone marrow transplantation in past six months	*	*	*
Respiratory cancer	211 (0.15)	74 (2.06)	38 (1.06)
Radiotherapy in the past six months	181 (0.12)	54 (1.50)	106 (2.94)
Solid organ transplantation	49 (0.03)	7 (0.19)	46 (1.28)
Chronic obstructive pulmonary disease	1889 (1.30)	580 (16.11)	331 (9.19)
Pulmonary hypertension	87 (0.06)	31 (0.86)	23 (0.64)
Coronary heart disease	3312 (2.28)	935 (25.97)	290 (8.06)
Stroke	2020 (1.39)	675 (18.75)	175 (4.86)
Atrial fibrillation	2457 (1.69)	955 (26.53)	234 (6.50)
Congestive cardiac failure	1251 (0.86)	581 (16.14)	159 (4.42)
Venous thromboembolism	2269 (1.56)	392 (10.89)	236 (6.56)
Peripheral vascular disease	586 (0.40)	235 (6.53)	56 (1.56)
Congenital heart disease	821 (0.56)	13 (0.36)	45 (1.25)
Dementia	1630 (1.12)	1100 (30.56)	103 (2.86)
Parkinson’s disease	246 (0.17)	129 (3.58)	16 (0.44)
Epilepsy	1911 (1.31)	106 (2.94)	103 (2.86)
Rare neurological conditions	465 (0.32)	27 (0.75)	386 (10.72)
Cerebral palsy	163 (0.11)	2 (0.06)	8 (0.22)
Osteoporotic fracture	5429 (3.73)	731 (20.31)	282 (7.83)
Rheumatoid arthritis or systemic lupus erythematosus	3310 (2.28)	348 (9.67)	365 (10.14)
Cirrhosis	241 (0.17)	40 (1.11)	139 (3.86)
Bipolar disorder or schizophrenia	1392 (0.96)	58 (1.61)	42 (1.17)
Inflammatory bowel disease	1600 (1.10)	57 (1.58)	280 (7.78)
Sickle cell disease, HIV, or severe combined immunodeficiency	383 (0.26)	12 (0.33)	349 (9.69)
Type 1 diabetes	844 (0.58)	16 (0.44)	56 (1.56)
Type 2 diabetes	6322 (4.35)	997 (27.69)	473 (13.14)

Data are number (%) of participants unless stated otherwise. SD=standard deviation.

Ethnic groups based on categories in the 2021 census of England and Wales (www.ethnicity-facts-figures.service.gov.uk/style-guide/ethnic-groups).

* Cells with counts <5 suppressed.

Comparing QCOVID2 and QCOVID4, 2962 people were in the high risk groups on both measures (287 covid-19 related deaths), 638 were high risk on QCOVID2 and low risk on QCOVID4 (28 covid-19 related deaths), and 638 were high risk on QCOVID4 and low risk on QCOVID2 (46 covid-19 related deaths). Overall, we found 28 more covid-19 related deaths (6.1% of the total) which would be correctly identified in the QCOVID4 high risk group compared with the QCOVID2 high risk group.

**Fig 9:**
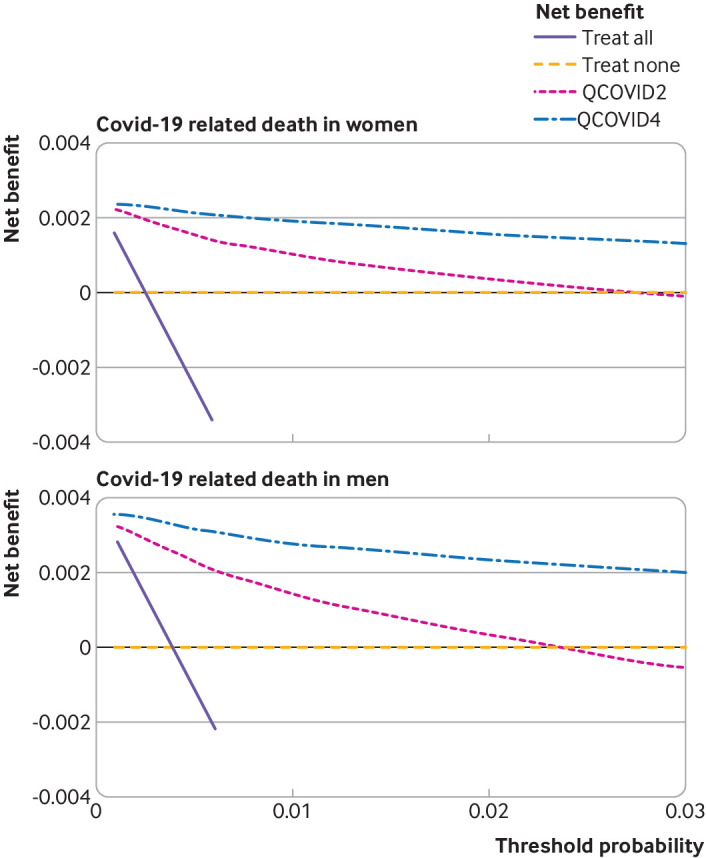
Decision curve analysis for risk of covid-19 related death showing the net benefit of using QCOVID4 compared with QCOVID2. Both algorithms were also compared with strategies of treating all patients or treating none

**Fig 10:**
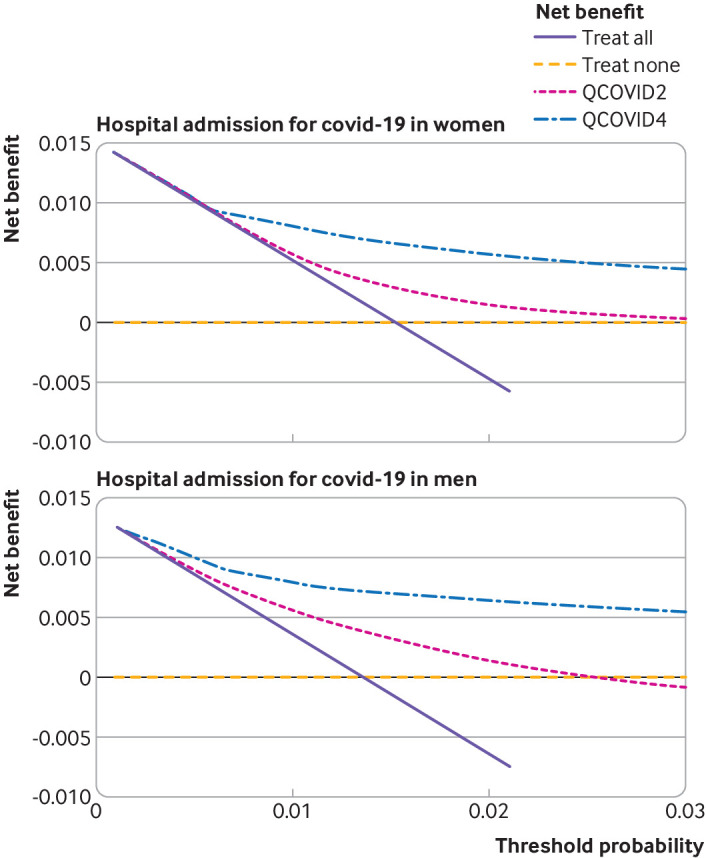
Decision curve analysis for risk of hospital admission for covid-19 showing the net benefit of using QCOVID4 compared with QCOVID2. Both algorithms were also compared with strategies of treating all patients or treating none

Figure 9 and figure 10 show the decision analysis curves which indicated the improved net benefit of using QCOVID4 compared with QCOVID2, and of both algorithms compared with strategies of treating all patients or treating none. Supplementary figure 5 shows the decision analysis curves by geographical region. These curves also indicate the potential clinical use of the models despite some lower net benefit in some of the regional subgroups.

## Discussion

### Principal findings

We have developed and validated a new QCOVID model (QCOVID4) based on data recorded during the period when the omicron variant of the SARS-CoV-2 virus was the predominant strain in England. QCOVID4 more accurately identified individuals at highest levels of absolute risk for targeted interventions than the conditions based approach adopted by NHS Digital, which is based on the relative risks of a list of medical conditions. QCOVID4 was also more accurate and had a higher net benefit than our earlier QCOVID2 algorithm at identifying high risk patients and had substantially better calibration in the current population. This finding likely reflects the largely vaccinated population and the lower virulence of the omicron compared with the alpha variant of the SARS-CoV-2 virus.

We also compared discrimination with earlier versions of the QCOVID algorithms on this dataset. The earlier QCOVID models were developed in the first wave of the original variant (QCOVID1) and second waves of the alpha and delta variants of the SARS-CoV-2 virus (QCOVID2). Overall, the factors associated with an increased risk in earlier models^1^ ^6^ were still associated with an increased risk in the QCOVID4 model, apart from residence, asthma, rare pulmonary conditions, cerebral palsy, and congenital heart disease. Another exception was ethnic group where the previously increased risks, particularly associated with South Asian and black African and Caribbean ethnic groups for covid-19 related death in QCOVID1^1^ and QCOVID2^6^ were no longer apparent in QCOVID4 although these results should be interpreted with caution because of possible differential testing rates. However, we saw a residual increased risk for Pakistani, Bangladeshi, and other Asian groups for admission to hospital for covid-19 compared with the white group for both men and women and for black African women, after adjustment for age, deprivation score, comorbidity, and vaccination status.

We have shown that infection with the SARS-CoV-2 virus before the study period was associated with about a 50% lower risk of covid-19 related death in both men and women. This finding was independent of age, ethnic group, deprivation score, comorbidity, and vaccination status. Similarly, we saw a dose dependent reduction in the risk of covid-19 related mortality in men and women after covid-19 vaccination, with each subsequent dose associated with a lower risk.

The validation showed that all three models (QCOVID4, QCOVID1, and QCOVID2) have high levels of discrimination and explained variation for covid-19 related death in this dataset. The QCOVID4 model has substantially improved discrimination and explained variation for predicting the risk of hospital admission for covid-19. Of those identified by NHS Digital as a high risk group for targeted treatments (2.5% of total), only 14% were also included in an equivalently sized high risk group with QCOVID4. The covid-19 related death rate in the QCOVID4 high risk group was three times higher than that in the NHS Digital cohort, supporting the face validity of QCOVID4. This difference was not explained by the use of treatment interventions, which was low in both groups. Similarly, QCOVID2 was more accurate in identifying high risk patients than the NHS Digital approach.

The validation results also showed that the QCOVID4 model was well calibrated to the current contemporaneous validation dataset with a small degree of underprediction of risk for covid-19 related death and overprediction at the highest levels of risk for hospital admission for covid-19. QCOVID1 was developed in a general population to estimate the combined risk of catching and dying from covid-19 because of lack of testing data available in the first wave of the pandemic and so estimates of absolute risk would not be valid for prediction of covid-19 related death in people with a positive test result for SARS-CoV-2. Overprediction was associated with QCOVID2, which likely mainly reflects higher levels of vaccination, better treatments, and differences in the variant type, with the omicron variant of the virus now generally considered to be less severe than earlier variants.^7^ ^30^ Taken together, QCOVID1 and QCOVID2 have acceptable ongoing use for ranking those at highest risk of death for interventions but are less robust for predicting the absolute risk of each outcome than QCOVID4.

### Strengths and limitations of the study

Our study had major strengths and some limitations. Limitations included specific problems related to covid-19 along with other strengths and limitations characteristic of widely used clinical risk prediction algorithms developed with the QResearch database.^31^ ^32^ ^33^ Key strengths included the use of large representative population based contemporaneous data sources which have been used to develop other widely used risk prediction tools,^31^ ^32^ an extensive set of diagnostic predictors from electronic healthcare records, prospective recording of outcomes and their determination with multiple national level database linkage, lack of selection, recall, and respondent biases, and robust statistical analysis. The size of the dataset makes overfitting unlikely.^34^ We used non-linear terms to model associations with body mass index and age, and multiple imputation to handle missing data. Finally, the update to the algorithm is a strength; prediction algorithms are seldom updated, despite changes in the natural history of a disease and the introduction of interventions.

Limitations included the relatively small numbers of events in some of the subgroups, which is an inevitable consequence of undertaking an analysis of multiple subgroups during a relatively short wave of a pandemic. Although we have accounted for many risk factors for severe covid-19 outcomes, additional risks might arise from rare medical conditions or other factors associated with infection, such as occupation, that are poorly recorded in general practice or hospital records. We could not separately identify those patients admitted to hospital because of SARS-CoV-2 infection from those admitted for other reasons where a coincidental infection was present. We used the first positive test results during the study period rather than account for repeat infections, although our study window was short (maximum 3.5 months) so the number of new reinfections should be small. Also, our study did not look at outcomes related to the recent omicron BA.4/BA.5 wave in England which was identified as a variant of concern on 18May 2022.

Although we have reported a validation based on practices from QResearch, these practices were separate from those used to develop the model. Previously we have used this approach to develop and validate other widely used prediction models. When these models have been validated on data from different clinical computer systems, the results have been similar.^35^ ^36^ ^37^ Work has now been completed to evaluate earlier QCOVID models in external datasets, including the English national dataset hosted by the Office of National Statistics,^2^ and Scotland^4^ and Wales^3^ data, and these evaluations also showed similar levels of performance to the validation in QResearch practices.

### Implications for clinical practice, policy, and research

The use of a risk prediction model depends on the purpose for which it has been designed, the setting in which it has been developed, and where it might be used. Our model provides a mechanism to estimate risk if a person has a positive test result and might require treatment. The speed at which new SARS-CoV-2 variants of concern have emerged and become dominant means that prediction models could be out of date almost as soon as they have been developed and implemented. This study, with its comparisons of algorithms developed over the past three major waves of the pandemic, each associated with different variant types, provides evidence that the discrimination of the QCOVID1 and QCOVID2 algorithms remains good. This finding means that the latest QCOVID algorithm is likely to be effective for risk stratifying or ranking individuals for interventions, even potentially when a new variant emerges, although continual monitoring for changes in risk factors is warranted. Algorithms might need to be recalibrated before being used to calculate absolute risks in settings or time periods with different rates for covid-19 related death or hospital admission for covid-19. QCOVID4 can be temporally recalibrated with a similar method to that used to temporally recalibrate QCOVID1.^1^

### Conclusion

The QCOVID4 algorithm, developed based on data from the period when the omicron variant of the SARS-CoV-2 virus was predominant in England, performed well in identifying those at highest risk of severe covid-19 outcomes. This approach can be used to risk stratify patients for intervention (such as covid-19 treatments) and inform clinical decision making on individualised risk management. This strategy could be more effective than an approach based on relative risks of individual medical conditions.

**What is already known on this topic**

The QCOVID risk assessment algorithm for predicting risk of covid-19 related death or hospital admission for covid-19 based on individual characteristics has been used in England to identify people at high risk of severe covid-19 outcomes
Treatments for covid-19 (monoclonal antibodies and antivirals) are available but need to be targeted to those at highest risk of severe outcomes

**What this study adds**

The QCOVID4 risk algorithm, based on data from the period when the omicron variant was predominant in England, now includes number of vaccination doses and previous SARS-CoV-2 infection
QCOVID4 performed well both for ranking individuals (discrimination) and predicting levels of absolute risk (calibration), and can be used for targeting covid-19 treatments as well as individualised risk assessment
QCOVID4 more accurately identified individuals at the highest levels of absolute risk than the approach for targeted interventions adopted by NHS Digital based on a list of medical conditions with increased relative risks

## Data Availability

To guarantee the confidentiality of personal and health information, only the authors have had access to the data during the study in accordance with the relevant licence agreements. Access to the QResearch data are according to the information on the QResearch website (www.qresearch.org). The full model, model coefficients, functional form, and cumulative incidence function are published on the www.qcovid.org website (https://bmj2022.qcovid.org).

## Web extra

Extra material supplied by authors

Web appendix: Supplementary tables and figures

## Notes

Contributors: Study conceptualisation was led by JH-C and CACC. JH-C specified the data, and organised data approvals and data linkage. JH-C and CACC designed the statistical analysis plan and undertook the analyses. JH-C wrote the first draft of the paper. AS, KK, and JSNVT contributed to development of study ideas, analysis plan, and provided critical comments on the manuscript. The Office of the Chief Medical Officer contributed to the development of the study question and facilitated access to relevant national datasets. JH-C is the guarantor. All authors contributed to the interpretation of the results and revision of the manuscript and approved the final version of the manuscript. The corresponding author attests that all listed authors meet authorship criteria and that no others meeting the criteria have been omitted.

JH-C had full access to all data in the study and takes responsibility for the integrity of the data and the accuracy of the data analysis. JH-C is the guarantor for the study and affirms that the manuscript is an honest, accurate, and transparent account of the study reported; that no important aspects of the study have been omitted and that any discrepancies from the study as planned have been explained.

Dissemination to participants and related patient and public communities: Patients have advised on disseminating the results, including the development of infographics (see supplementary fig 6) and its translation into different languages.

Provenance and peer review: Not commissioned; externally peer reviewed.
