# Supplementary material for: Risk prediction of covid-19 related death or hospital admission in adults testing positive for SARS-CoV-2 infection during the omicron wave in England (QCOVID4): cohort study
Source: BMJ. 2023 Jun 21;381:e072976. doi: 10.1136/bmj-2022-072976 (PMC10282241; doi:10.1136/bmj-2022-072976)

QCOVID 4 - Predicting risk of death or hospitalisation from COVID-19 in adults testing positive for SARS-CoV-2 infection during the Omicron wave in England: Cohort study

SUPPLEMENTARY TABLES AND FIGURES

Julia Hippisley-Cox, *Professor of Clinical Epidemiology & General Practice, University of Oxford*[1]
Kamlesh Khunti, *Professor of Primary Care Diabetes & Vascular Medicine [3]*
Aziz Sheikh, *Professor of Primary Care Research & Development and Director [4]*
Jonathan S Nguyen-Van-Tam, *Professor of Health Protection [2]*
Carol AC Coupland, *Senior Research Fellow* at the University of Oxford [1] and *Professor of Medical Statistics in Primary Care at the University of Nottingham[2]*

Institutions
1 Nuffield Department of Primary Health Care Sciences, University of Oxford
2 Lifespan and Population Health Unit, School of Medicine, University of Nottingham, Nottingham, UK
3 Diabetes Research Centre, University of Leicester, UK
4 Usher Institute, University of Edinburgh, Edinburgh, UK

SUPPLEMENTARY TABLE 1 Baseline characteristics of the validation cohort of patients with a SARS-CoV-2 positive test, COVID-19 death and COVID-19 admission

	total population	SARS-CoV-2 positive	COVID-19 death	COVID-19 admission
total	1,064,255	145,397	461	2,124
males (%)	531631 (49.95)	62305 (42.85)	240 (52.06)	846 (39.83)
mean age (SD)	47.85 (18.49)	42.96 (16.41)	81.30 (11.87)	55.79 (22.10)
White	699679 (65.74)	101478 (69.79)	356 (77.22)	1474 (69.40)
Indian	23101 (2.17)	2897 (1.99)	*	33 (1.55)
Pakistani	26677 (2.51)	2291 (1.58)	*	59 (2.78)
Bangladeshi	16650 (1.56)	1550 (1.07)	*	29 (1.37)
Other Asian	16571 (1.56)	2046 (1.41)	*	26 (1.22)
Caribbean	8336 (0.78)	959 (0.66)	5 (1.08)	33 (1.55)
Black African	26218 (2.46)	2743 (1.89)	*	56 (2.64)
Chinese	9650 (0.91)	902 (0.62)	*	11 (0.52)
Other ethnic group	39788 (3.74)	4931 (3.39)	6 (1.30)	77 (3.63)
Ethnicity not recorded	197585 (18.57)	25600 (17.61)	80 (17.35)	326 (15.35)
1 (most affluent)	273276 (25.68)	38571 (26.53)	125 (27.11)	478 (22.50)
2	239009 (22.46)	34309 (23.60)	128 (27.77)	420 (19.77)
3	199585 (18.75)	28114 (19.34)	97 (21.04)	417 (19.63)
4	175259 (16.47)	22214 (15.28)	62 (13.45)	389 (18.31)
5 (most deprived)	163433 (15.36)	19639 (13.51)	48 (10.41)	382 (17.98)
6 (not recorded)	13693 (1.29)	2550 (1.75)	*	38 (1.79)
East Midlands	21799 (2.05)	3039 (2.09)	7 (1.52)	23 (1.08)
East of England	56069 (5.27)	8358 (5.75)	21 (4.56)	106 (4.99)
London	235847 (22.16)	30999 (21.32)	53 (11.50)	453 (21.33)
North East	9949 (0.93)	1573 (1.08)	8 (1.74)	21 (0.99)
North West	252973 (23.77)	35672 (24.53)	136 (29.50)	591 (27.82)
South Central	46299 (4.35)	6628 (4.56)	19 (4.12)	94 (4.43)
South East	164012 (15.41)	23201 (15.96)	75 (16.27)	273 (12.85)
South West	95885 (9.01)	13251 (9.11)	36 (7.81)	183 (8.62)
West Midlands	160269 (15.06)	19640 (13.51)	98 (21.26)	334 (15.73)
Yorkshire & Humber	21153 (1.99)	3036 (2.09)	8 (1.74)	46 (2.17)
neither	1054342 (99.07)	143343 (98.59)	350 (75.92)	1989 (93.64)
Care home	8137 (0.76)	1903 (1.31)	110 (23.86)	125 (5.89)
Homeless	1776 (0.17)	151 (0.10)	*	10 (0.47)
BMI < 18.5	24788 (2.33)	3443 (2.37)	37 (8.03)	69 (3.25)
BMI 18.5-24.99	302741 (28.45)	43886 (30.18)	140 (30.37)	571 (26.88)
BMI 25-29.99	260970 (24.52)	35152 (24.18)	108 (23.43)	517 (24.34)

BMI 30-34.99	123125 (11.57)	16520 (11.36)	57 (12.36)	324 (15.25)
BMI 35+	47646 (4.48)	6825 (4.69)	22 (4.77)	128 (6.03)
BMI 40+	24866 (2.34)	3655 (2.51)	11 (2.39)	51 (2.40)
BMI not recorded	280119 (26.32)	35916 (24.70)	86 (18.66)	464 (21.85)
no CKD	1020120 (95.85)	141747 (97.49)	293 (63.56)	1830 (86.16)
CKD5 only	1226 (0.12)	164 (0.11)	7 (1.52)	28 (1.32)
CKD5 with dialysis	369 (0.03)	53 (0.04)	*	*
CKD5 with transplant	592 (0.06)	113 (0.08)	*	27 (1.27)
no learning disability	1044039 (98.10)	142758 (98.18)	454 (98.48)	2059 (96.94)
Learning disability	19731 (1.85)	2553 (1.76)	6 (1.30)	62 (2.92)
Downs	485 (0.05)	86 (0.06)	*	*
No chemo in last 12	1060048 (99.60)	144888 (99.65)	432 (93.71)	2065 (97.22)
Chemo group A	1945 (0.18)	207 (0.14)	9 (1.95)	15 (0.71)
chemo group B	2154 (0.20)	284 (0.20)	19 (4.12)	42 (1.98)
chemo group C	108 (0.01)	18 (0.01)	*	*
no type1 diabetes	1059034 (99.51)	144553 (99.42)	459 (99.57)	2094 (98.59)
type 1 HBA<=59	1515 (0.14)	275 (0.19)	*	9 (0.42)
type1 HBA1C 59+	3056 (0.29)	463 (0.32)	*	18 (0.85)
type 1 HBA1C no record	650 (0.06)	106 (0.07)	*	*
no type2 diabetes	992924 (93.30)	139075 (95.65)	340 (73.75)	1839 (86.58)
type 2 HBA<=59	40138 (3.77)	3620 (2.49)	76 (16.49)	152 (7.16)
type2 HBA1C 59+	22464 (2.11)	1932 (1.33)	33 (7.16)	99 (4.66)
type 2 HBA1C no record	8729 (0.82)	770 (0.53)	12 (2.60)	34 (1.60)
no covid vaccine doses	181718 (17.07)	15725 (10.82)	36 (7.81)	416 (19.59)
1 dose	31020 (2.91)	4985 (3.43)	12 (2.60)	89 (4.19)
2 doses	169936 (15.97)	39637 (27.26)	87 (18.87)	418 (19.68)
3 doses	587386 (55.19)	84272 (57.96)	317 (68.76)	1136 (53.48)
4 doses	94195 (8.85)	778 (0.54)	9 (1.95)	65 (3.06)
SARS-CoV-2 infection prior to study	147803 (13.89)	16014 (11.01)	27 (5.86)	134 (6.31)
Blood cancer	8393 (0.79)	998 (0.69)	38 (8.24)	91 (4.28)
Bone marrow transplant in previous 6 months	38 (0.00)	*	*	*
Respiratory cancer	2258 (0.21)	211 (0.15)	14 (3.04)	16 (0.75)
radiotherapy in last 6/12	1401 (0.13)	181 (0.12)	8 (1.74)	25 (1.18)
solid organ transplant	233 (0.02)	49 (0.03)	1 (0.22)	9 (0.42)
COPD	23367 (2.20)	1889 (1.30)	72 (15.62)	180 (8.47)
Asthma	146396 (13.76)	23767 (16.35)	76 (16.49)	378 (17.80)

rare pulmonary conditions	6016 (0.57)	601 (0.41)	17 (3.69)	42 (1.98)
pulmonary hypertension	937 (0.09)	87 (0.06)	7 (1.52)	6 (0.28)
coronary heart disease	37471 (3.52)	3312 (2.28)	104 (22.56)	230 (10.83)
stroke	23218 (2.18)	2020 (1.39)	89 (19.31)	141 (6.64)
atrial fibrillation	26343 (2.48)	2457 (1.69)	135 (29.28)	163 (7.67)
congestive cardiac failure	13976 (1.31)	1251 (0.86)	69 (14.97)	109 (5.13)
VTE	20761 (1.95)	2269 (1.56)	46 (9.98)	127 (5.98)
PVD	7500 (0.70)	586 (0.40)	32 (6.94)	47 (2.21)
congenital heart disease	5234 (0.49)	821 (0.56)	*	13 (0.61)
dementia	11518 (1.08)	1630 (1.12)	119 (25.81)	160 (7.53)
Parkinson's disease	2550 (0.24)	246 (0.17)	23 (4.99)	24 (1.13)
epilepsy	14250 (1.34)	1911 (1.31)	12 (2.60)	64 (3.01)
rare neurological conditions	3344 (0.31)	465 (0.32)	7 (1.52)	47 (2.21)
cerebral palsy	1352 (0.13)	163 (0.11)	*	7 (0.33)
osteoporotic fracture	43404 (4.08)	5429 (3.73)	85 (18.44)	177 (8.33)
RA or SLE	28446 (2.67)	3310 (2.28)	43 (9.33)	153 (7.20)
cirrhosis	2413 (0.23)	241 (0.17)	6 (1.30)	23 (1.08)
bipolar disorder or schizophrenia	12029 (1.13)	1392 (0.96)	*	47 (2.21)
IBS	10167 (0.96)	1600 (1.10)	11 (2.39)	51 (2.40)
sickle cell disease, HIV or severe combined immunodeficiency	2682 (0.25)	383 (0.26)	*	17 (0.80)

SUPPLEMENTARY TABLE 2: Fully adjusted HR for COVID-19 death and admission in men and women summarising the results for vaccination dose and SARS-CoV-2 infection prior to study period. HR adjusted for age and BMI as well as variables shown in the separate Figures 1-4

	COVID-19 death		COVID-19 admission	
	women	men	women	men
No COVID vaccine	1	1	1	1
1 dose	0.64 (0.47, 0.87)	0.58 (0.43, 0.79)	0.67 (0.61, 0.74)	0.66 (0.58, 0.75)
2 doses	0.49 (0.41, 0.59)	0.50 (0.42, 0.60)	0.39 (0.36, 0.41)	0.40 (0.37, 0.43)
3 doses	0.20 (0.17, 0.24)	0.19 (0.16, 0.22)	0.25 (0.23, 0.26)	0.24 (0.23, 0.26)
4+ doses	0.14 (0.10, 0.20)	0.08 (0.06, 0.12)	0.41 (0.36, 0.47)	0.27 (0.23, 0.31)
prior SARS-CoV-2	0.55 (0.45, 0.67)	0.51(0.40, 0.64)	0.67 (0.63, 0.72)	0.61 (0.56, 0.68)

SUPPLEMENTARY TABLE 3: Performance of the QCOVID4 algorithms in the validation cohort in subgroups for age group and ethnicity.

	COVID-19 death		COVID-19 admission	
**statistic**	**females**	**males**	**females**	**males**
**<70 years**				
D statistic	2.96 (2.07 to 3.84)	2.56 (1.93 to 3.19)	2.91 (2.04 to 3.78)	2.42 (1.81 to 3.02)
Harrell's C	.915 (.856 to .973)	.894 (.846 to .942)	.945 (.918 to .972)	.904 (.863 to .945)
R2	67.6 (54.5 to 80.7)	61.1 (49.3 to 72.8)	66.9 (53.7 to 80.2)	58.3 (46.1 to 70.4)
Calibration slope	1.13 (.936 to 1.33)	1.29 (1.11 to 1.47)	1.08 (1.02 to 1.14)	1.12 (1.05 to 1.19)
**70-79 years**				
D statistic	2.63 (2.11 to 3.15)	2.06 (1.61 to 2.51)	1.94 (1.47 to 2.41)	1.73 (1.3 to 2.17)
Harrell's C	.88 (.831 to .929)	.833 (.777 to .889)	.817 (.765 to .869)	.79 (.733 to .848)
R2	62.3 (53 to 71.5)	50.3 (39.4 to 61.1)	47.3 (35.1 to 59.4)	41.7 (29.5 to 54)
Calibration slope	1.31 (1.1 to 1.53)	1.15 (.94 to 1.36)	.939 (.805 to 1.07)	1.12 (.971 to 1.26)
**80+ years**				
D statistic	1.12 (.79 to 1.46)	1.17 (.905 to 1.44)	.941 (0.68 to 1.2)	1.04 (.779 to 1.29)
Harrell's C	.673 (.63 to .717)	.713 (.675 to .752)	.664 (.621 to .707)	.698 (.659 to .736)
R2	23.2 (12.6 to 33.8)	24.8 (16.2 to 33.3)	17.4 (9.46 to 25.4)	20.4 (12.4 to 28.5)
Calibration slope	.731 (.516 to .947)	.702 (.542 to .861)	.567 (.424 to .709)	.5 (.343 to .657)
**White**				
D statistic	3.68 (3.44 to 3.91)	3.64 (3.4 to 3.87)	3.36 (3.12 to 3.59)	3.44 (3.22 to 3.66)
Harrell's C	.963 (.948 to .979)	.969 (.96 to .978)	.964 (.955 to .973)	.968 (.961 to .976)
R2	76.4 (74.1 to 78.7)	76 (73.6 to 78.3)	72.9 (70.2 to 75.7)	73.9 (71.4 to 76.4)
Calibration slope	1.02 (.943 to 1.1)	1.01 (.934 to 1.08)	.991 (.948 to 1.03)	.986 (.941 to 1.03)
**Indian**				
D statistic	*	4.38 (1.16 to 7.61)	*	3.61 (.811 to 6.41)
Harrell's C	.975 (.948 to 1)	.994 (.99 to .998)	.971 (.951 to .991)	.989 (.98 to .998)
R2	*	81.1 (57 to 105)	*	74.6 (43.7 to 106)
Calibration slope	*	1.15 (.385 to 1.91)	.635 (.103 to 1.17)	1.17 (.871 to 1.46)
**Pakistani**				
D statistic	3.5 (1.56 to 5.44)	*	2.76 (.939 to 4.57)	*
Harrell's C	.971 (.938 to 1)	.989 (.983 to .995)	.867 (.668 to 1.07)	.981 (.974 to .989)
R2	74.5 (53.5 to 95.5)	*	64.4 (34 to 94.7)	*
Calibration slope	.753 (.378 to 1.13)	.965 (.06 to 1.87)	1.05 (.78 to 1.31)	1.33 (1.01 to 1.66)
**Bangladeshi**				
D statistic	4.44 (.539 to 8.35)	4.79 (.392 to 9.19)	4.31 (.342 to 8.27)	*
Harrell's C	.992 (.987 to .998)	.994 (.989 to .999)	.984 (.977 to .992)	*
R2	82.4 (57.4 to 107)	84.6 (60.4 to 109)	81.2 (53.4 to 109)	97.6 (81.4 to 114)
Calibration slope	.981 (.341 to 1.62)	*	1.08 (.76 to 1.4)	1.54 (1.09 to 1.98)

**Other Asian**				
D statistic	*	3.88 (1.37 to 6.39)	*	5.57 (1.82 to 9.32)
Harrell's C	*	.974 (.951 to .998)	*	.981 (.956 to 1.01)
R2	*	78.1 (55.1 to 101)	*	87.8 (74 to 102)
Calibration slope	1.14 (.814 to 1.47)	.877 (.297 to 1.46)	1.09 (.819 to 1.37)	1.1 (.564 to 1.64)
**Caribbean**				
D statistic	5.6 (.00527 to 11.2)	4.64 (2.12 to 7.17)	*	3.81 (1.91 to 5.71)
Harrell's C	.997 (.993 to 1)	.969 (.947 to .991)	.984 (.974 to .993)	.965 (.944 to .986)
R2	87.9 (68.1 to 108)	83.6 (68.9 to 98.3)	76.3 (41.1 to 112)	77.5 (60.4 to 94.7)
Calibration slope	1.32 (-.258 to 2.91)	1.24 (.506 to 1.97)	.963 (.657 to 1.27)	1.04 (.727 to 1.36)
**Black African**				
D statistic	4.93 (1.77 to 8.1)	41.6 (-196 to 279)	4.34 (1.5 to 7.18)	5.69 (1.06 to 10.3)
Harrell's C	.995 (.992 to .998)	.995 (.989 to 1)	.993 (.988 to .997)	.998 (.995 to 1)
R2	85.3 (69.2 to 101)	92.9 (76.6 to 109)	81.8 (62.4 to 101)	88.4 (71.6 to 105)
Calibration slope	1.49 (.391 to 2.6)	2.07 (.107 to 4.03)	.971 (.765 to 1.18)	.784 (.35 to 1.22)
**Chinese**				
D statistic	*	*	*	*
Harrell's C	*	.993 (.985 to 1)	*	.988 (.978 to .999)
R2	51.4 (-90.9 to 194)	76.9 (23.7 to 130)	*	76 (17.5 to 135)
Calibration slope	1.21 (-.158 to 2.58)	.968 (.466 to 1.47)	1.15 (.636 to 1.66)	1.31 (.847 to 1.77)
**Other**				
D statistic	5.74 (2.15 to 9.33)	3.17 (1.57 to 4.78)	5.25 (1.8 to 8.7)	3.2 (1.53 to 4.87)
Harrell's C	.994 (.985 to 1)	.945 (.903 to .986)	.992 (.98 to 1)	.929 (.866 to .992)
R2	88.6 (76.3 to 101)	70.5 (49.2 to 91.8)	86.5 (71.9 to 101)	70.7 (48.7 to 92.7)
Calibration slope	1.2 (.411 to 1.99)	.793 (.478 to 1.11)	1.2 (.941 to 1.47)	1.19 (.967 to 1.42)

*counts too low for analysis

SUPPLEMENTARY TABLE 4 Performance of the QCOVID4 algorithms in the validation cohort in subgroups by geographical region

		COVID-19 death		COVID-19 admission	
region	statistic	females	males	females	males
East Midlands	D statistic	4.84 (2.7 to 6.97)	*	4.13 (2.39 to 5.88)	*
	Harrell's C	.98 (.972 to .988)	.999 (.998 to 1)	.985 (.979 to .99)	.996 (.994 to .999)
	R2	84.8 (73.5 to 96.2)	91.2 (78.1 to 104)	80.3 (67 to 93.6)	92.8 (103 to 82.6)
	slope	1.09 (.483 to 1.7)	1.55 (.375 to 2.73)	.964 (.563 to 1.37)	*
East of England	D statistic	4.01 (2.74 to 5.28)	3.97 (2.92 to 5.03)	3.85 (2.66 to 5.04)	3.61 (2.6 to 4.62)
	Harrell's C	.983 (.979 to .987)	.979 (.974 to .985)	.984 (.981 to .988)	.98 (.976 to .984)
	R2	79.3 (68.9 to 89.7)	79 (70.2 to 87.8)	78 (67.4 to 88.6)	75.7 (65.4 to 86)
	slope	1.06 (.654 to 1.47)	1.15 (.794 to 1.5)	1.09 (.943 to 1.23)	1.14 (.946 to 1.34)
London	D statistic	3.93 (3.28 to 4.57)	3.85 (3.17 to 4.53)	3.21 (2.6 to 3.83)	3.84 (3.15 to 4.54)
	Harrell's C	.949 (.925 to .974)	.961 (.94 to .981)	.931 (.905 to .957)	.959 (.935 to .983)
	R2	78.6 (73.1 to 84.1)	78 (71.9 to 84.1)	71.1 (63.2 to 79)	77.9 (71.7 to 84.1)
	slope	.972 (.797 to 1.15)	.99 (.806 to 1.17)	1.00 (.918 to 1.08)	1.14 (1.04 to 1.24)
North East	D statistic	4.98 (2.27 to 7.68)	4.67 (2.68 to 6.67)	5.14 (7.98 to 2.29)	4.39 (2.48 to 6.31)
	Harrell's C	.993 (.99 to .997)	.992 (.989 to .995)	.993 (.99 to .996)	.981 (.97 to .992)
	R2	85.5 (72.1 to 99)	83.9 (72.4 to 95.4)	86.3 (99.4 to 73.2)	82.2 (69.4 to 94.9)
	slope	1.6 (.466 to 2.73)	1.81 (.702 to 2.91)	1.35 (.881 to 1.82)	1.04 (.61 to 1.48)
North West	D statistic	3.65 (3.25 to 4.06)	3.72 (3.31 to 4.14)	3.39 (3 to 3.79)	3.44 (3.03 to 3.84)
	Harrell's C	.966 (.957 to .974)	.975 (.971 to .979)	.96 (.954 to .966)	.971 (.967 to .975)
	R2	76.1 (72 to 80.1)	76.8 (72.8 to 80.8)	73.3 (68.7 to 77.9)	73.8 (69.3 to 78.3)
	slope	.955 (.825 to 1.09)	1.03 (.9 to 1.17)	.971 (.898 to 1.04)	.94 (.862 to 1.02)
South Central	D statistic	5.12 (3.53 to 6.71)	3.55 (2.49 to 4.61)	4.41 (3.04 to 5.79)	4.12 (2.9 to 5.34)
	Harrell's C	.994 (.993 to .996)	.976 (.971 to .98)	.99 (.987 to .992)	.975 (.968 to .981)
	R2	86.2 (78.8 to 93.6)	75.1 (63.9 to 86.2)	82.3 (73.2 to 91.4)	80.2 (70.8 to 89.6)
	slope	1.45 (.86 to 2.05)	.954 (.646 to 1.26)	.838 (.627 to 1.05)	1.04 (.867 to 1.22)
South East	D statistic	3.76 (3.19 to 4.33)	3.56 (3.01 to 4.1)	3.44 (2.89 to 3.99)	3.45 (2.92 to 3.98)
	Harrell's C	.984 (.982 to .986)	.968 (.961 to .974)	.97 (.966 to .975)	.97 (.966 to .975)
	R2	77.1 (71.8 to 82.5)	75.1 (69.4 to 80.8)	73.9 (67.7 to 80.1)	74 (68 to 79.9)
	slope	1.09 (.886 to 1.28)	.969 (.808 to 1.13)	1.03 (.906 to 1.15)	.979 (.857 to 1.1)
South West	D statistic	3.97 (3.06 to 4.89)	3.84 (3.07 to 4.61)	3.92 (3.01 to 4.83)	3.59 (2.85 to 4.34)
	Harrell's C	.985 (.981 to .988)	.977 (.97 to .984)	.981 (.977 to .985)	.975 (.967 to .983)
	R2	79 (71.4 to 86.7)	77.9 (71 to 84.8)	78.6 (70.8 to 86.4)	75.5 (67.8 to 83.2)
	slope	1.17 (.817 to 1.53)	1.07 (.817 to 1.32)	.916 (.773 to 1.06)	1.05 (.917 to 1.18)
West Midlands	D statistic	3.54 (3.03 to 4.05)	3.66 (3.17 to 4.14)	3.4 (2.91 to 3.88)	3.49 (3.02 to 3.96)
	Harrell's C	.948 (.931 to .965)	.973 (.969 to .977)	.961 (.953 to .97)	.969 (.965 to .974)
	R2	75 (69.6 to 80.4)	76.1 (71.3 to 81)	73.3 (67.7 to 79)	74.4 (69.3 to 79.5)
	slope	.961 (.792 to 1.13)	.986 (.84 to 1.13)	1.07 (.959 to 1.18)	.992 (.882 to 1.1)
Yorks & Humber	D statistic	2.98 (.573 to 5.39)	2.17 (.835 to 3.5)	4.1 (1.34 to 6.86)	2.55 (1.17 to 3.92)
	Harrell's C	.946 (.92 to .972)	.806 (.719 to .893)	.944 (.91 to .979)	.873 (.832 to .914)
	R2	68 (32.8 to 103)	52.9 (22.2 to 83.5)	80.1 (58.6 to 102)	60.7 (35 to 86.5)
	slope	.751 (.165 to 1.34)	.463 (.0967 to .83)	.814 (.478 to 1.15)	.892 (.514 to 1.27)

SUPPLEMENTARY TABLE 5: comparison of 4 QCOVID risk models

	QCOVID1	QCOVID2	QCOVID3	QCOVID4
**Study period**	24 Jan 2020 to 30 April 2020	8 Dec 2020 to 21 June 2021	8 Dec 2020 to 21 June 2021	11 Dec 2021 to 30 June 2022
**Dominant strain SARS-CoV-2 infection**	original strain	alpha	alpha	omicron
**Base population**	population based	(a) population based and (b) test positive	(a) population based and (b) test positive	test positive
**Vaccination status**	unvaccinated population	unvaccinated	partially vaccinated (one or two doses)	largely vaccinated
**Outcomes predicted**	COVID-19 deaths and COVID-19 admissions	COVID-19 deaths and COVID-19 admissions	COVID-19 deaths and COVID-19 admissions	COVID-19 deaths and COVID-19 admissions
**Cohort follow up**	from 24 Jan 2020	from 8 Dec 2020	from 14 days after date of 1st vaccination	from date of 1st SARS-CoV-2 positive test in study period
**predictor variables**				
sex	**YES**	**YES**	**YES**	**YES**
age	**YES**	**YES**	**YES**	**YES**
body mass index	**YES**	**YES**	**YES**	**YES**
Townsend deprivation	**YES**	**YES**	**YES**	**YES**
Leukotriene	**YES**	**YES**	NO	**YES**
Long acting beta agonist	**YES**	**YES**	NO	**YES**
Atrial fibrillation	**YES**	**YES**	NO	**YES**
Congestive cardiac failure	**YES**	**YES**	**YES**	**YES**
Asthma	**YES**	**YES**	NO	NO
Blood cancer	**YES**	**YES**	**YES**	**YES**
Cerebral palsy	**YES**	**YES**	NO	NO
Coronary heart disease	**YES**	**YES**	**YES**	**YES**
Liver cirrhosis	**YES**	**YES**	**YES**	**YES**
Congenital heart disease	**YES**	**YES**	NO	NO
COPD	**YES**	**YES**	**YES**	**YES**
Dementia	**YES**	**YES**	**YES**	**YES**
Epilepsy	**YES**	**YES**	**YES**	**YES**
Osteoporotic fracture	**YES**	**YES**	NO	**YES**
Inflammatory bowel disease	NO	**YES**	NO	**YES**
Schizophrenia or bipolar depression	NO	**YES**	**YES**	**YES**

Schizophrenia or bipolar depression or depression	**YES**	NO	NO	NO
Rare neurological conditions	**YES**	**YES**	**YES**	**YES**
Parkinson's disease	**YES**	**YES**	**YES**	**YES**
Pulmonary hypertension	**YES**	**YES**	NO	**YES**
Rare pulmonary conditions	**YES**	**YES**	NO	NO
Peripheral vascular disease	**YES**	**YES**	**YES**	**YES**
Rheumatoid arthritis or SLE	**YES**	**YES**	NO	**YES**
Respiratory cancer	**YES**	**YES**	**YES**	**YES**
Sickle cell disease, HIV or severe combined immunodeficiency	**YES**	**YES**	YES**	**YES**
Stroke	**YES**	**YES**	**YES**	**YES**
Type 1 diabetes	**YES**	YES*	NO	**YES**
Type 2 diabetes	**YES**	YES*	YES*	**YES**
VTE	**YES**	**YES**	**YES**	**YES**
chemotherapy categories	**YES**	**YES**	**YES**	**YES**
Ethnicity	**YES**	**YES**	**YES**	**YES**
Housing status	**YES**	**YES**	**YES**	NO
Learning disabilities	**YES**	**YES**	**YES**	**YES**
Bone marrow or solid organ transplant	**YES**	**YES**	**YES**	**YES**
Radiotherapy in past 6 months	**YES**	**YES**	**YES**	**YES**
Solid organ transplant	**YES**	**YES**	**YES**	**YES**
Renal failure categories	**YES**	**YES**	**YES**	**YES**
prior infection	NO	NO	NO	**YES**
vaccination dose	NO	NO	2 doses vs 1 dose	0/1/2/3/4 doses
background infection rate	NO	**YES**	**YES**	NO

*HBA1C subdivided by value
** as three separate variables

SUPPLEMENTARY FIGURES

Supplementary Figure 1a Adjusted hazard ratio (95% CI) by age for risk of COVID-19 mortality

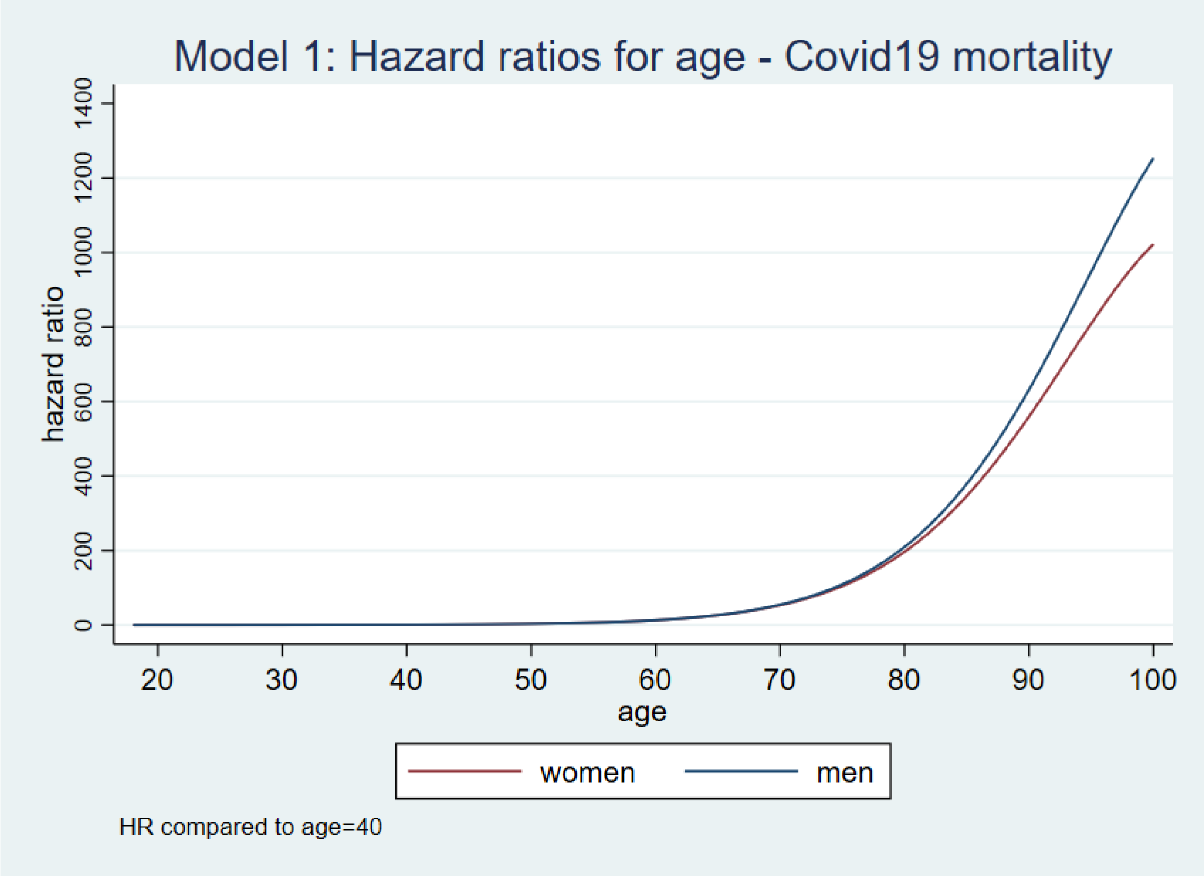

Supplementary Figure 1b Adjusted hazard ratio (95% CI) by BMI for COVID-19 mortality

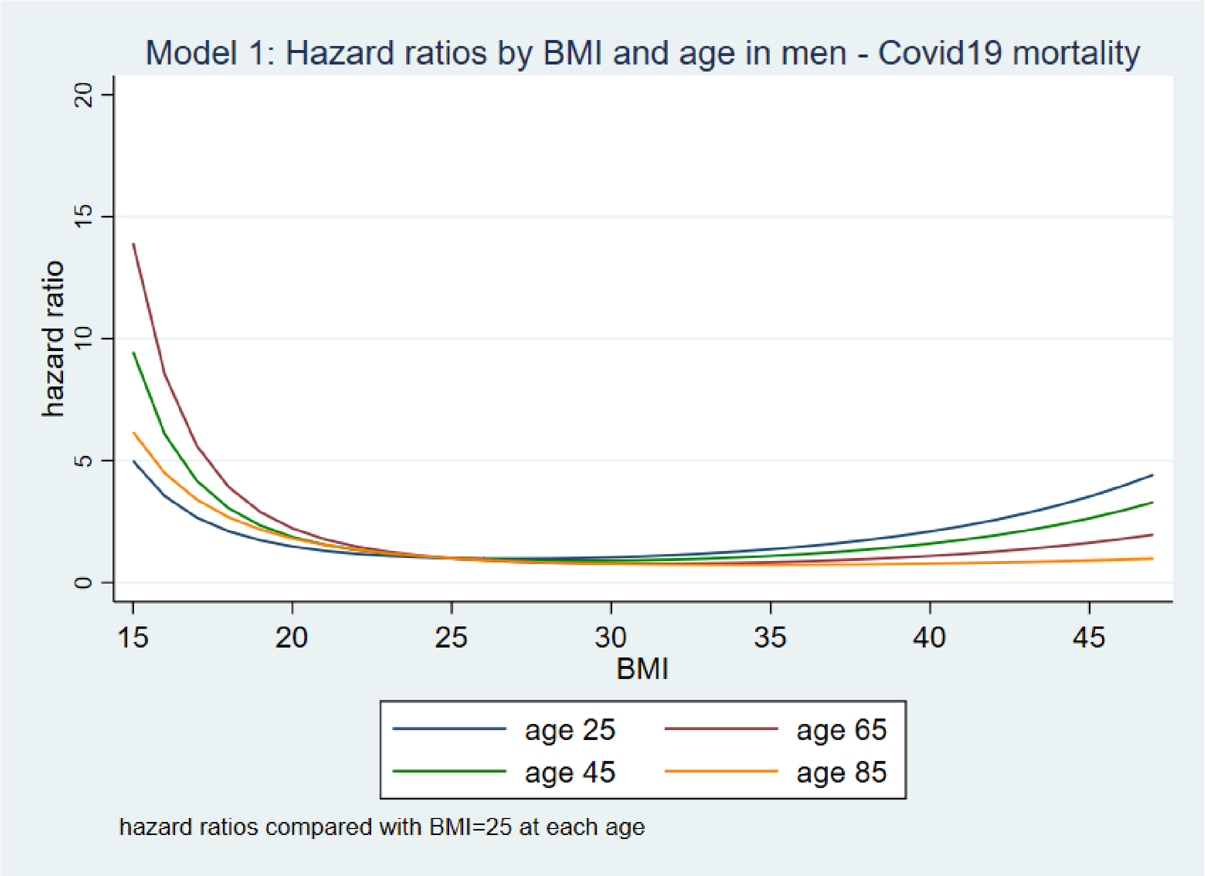

Supplementary Figure 1c Adjusted hazard ratio (95% CI) by age for risk of COVID-19 admission

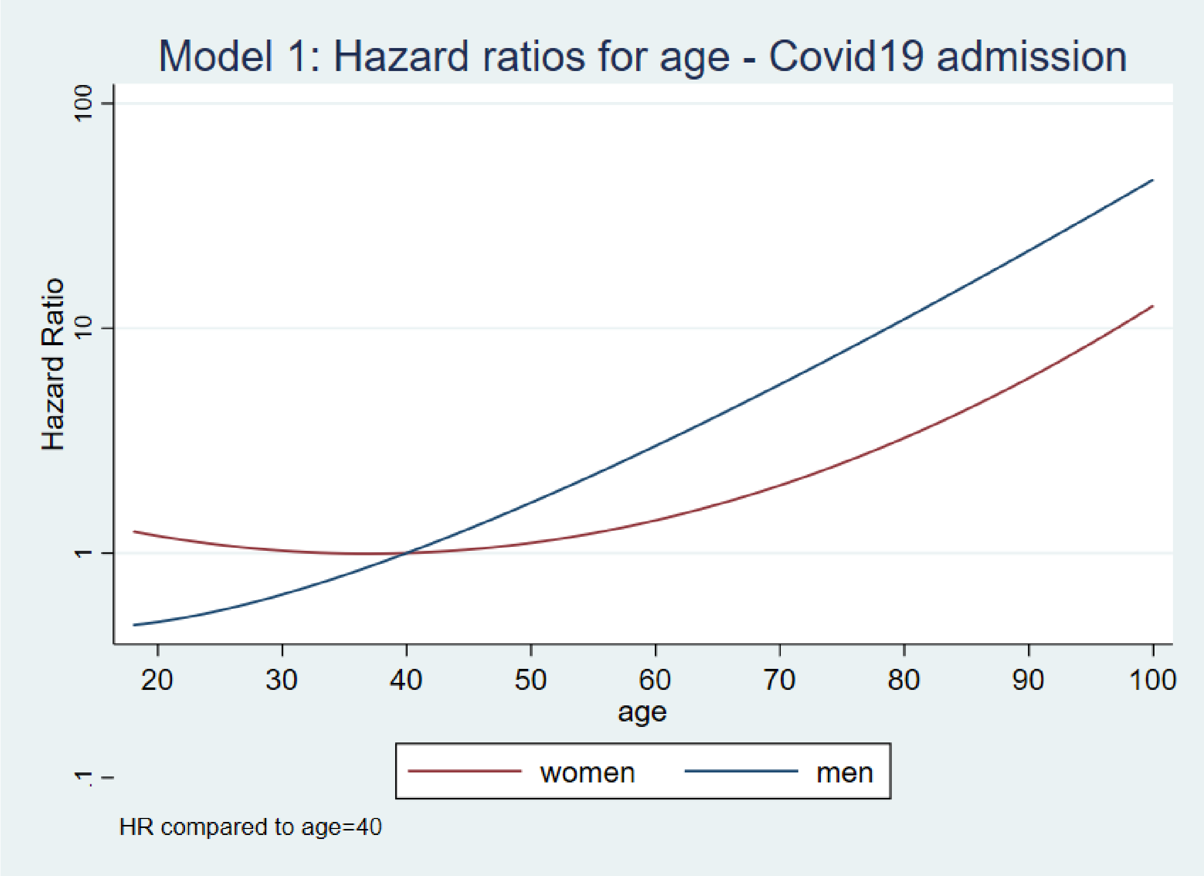

Supplementary Figure 1d Adjusted hazard ratio (95% CI) by BMI for COVID-19 admission

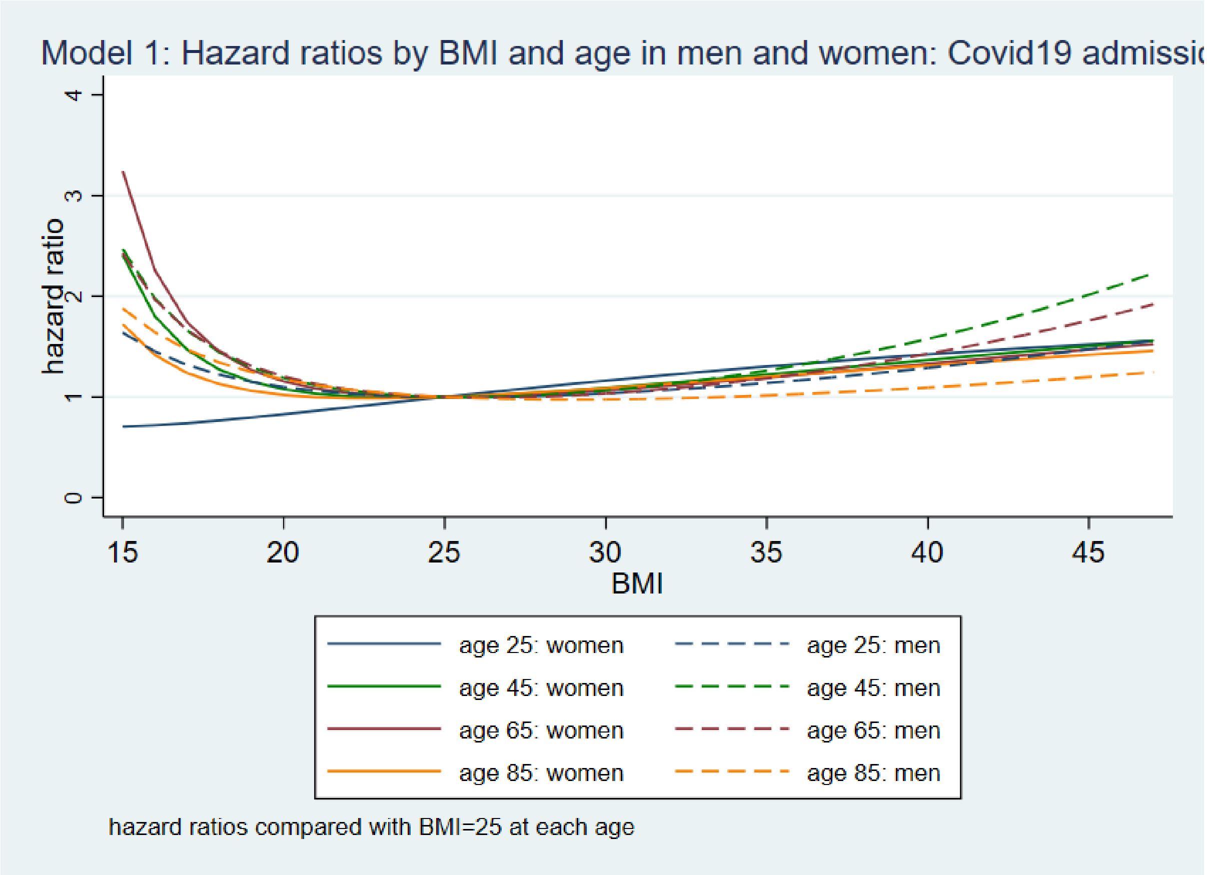

Supplementary Figure 2a Smoothed calibration plots for risk of death by ethnic group in women

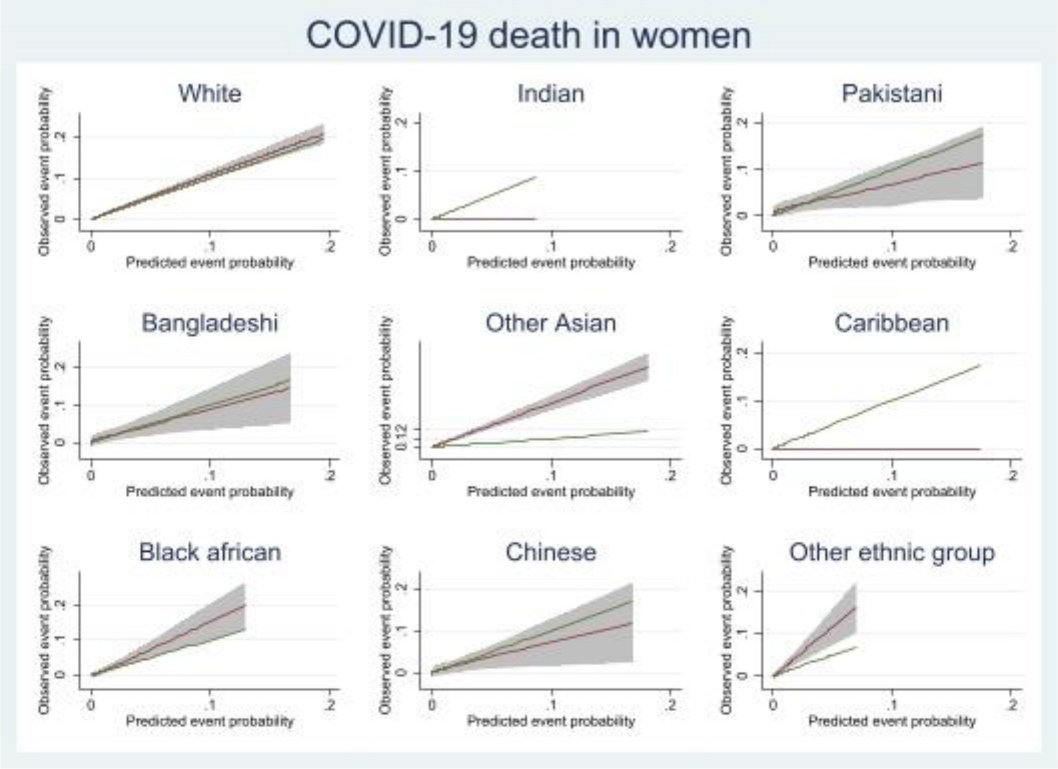

Supplementary Figure 2b Smoothed calibration plots for risk of death by ethnic group in men

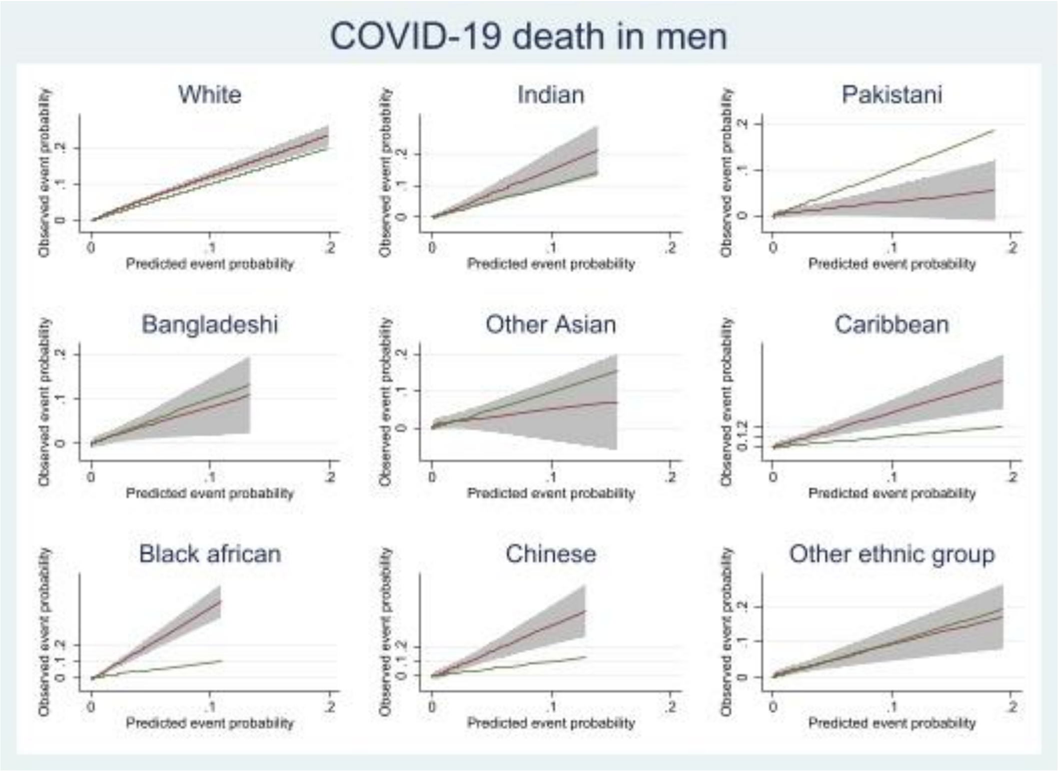

Supplementary Figure 2c Smoothed calibration plots for risk of admission by ethnic group in women

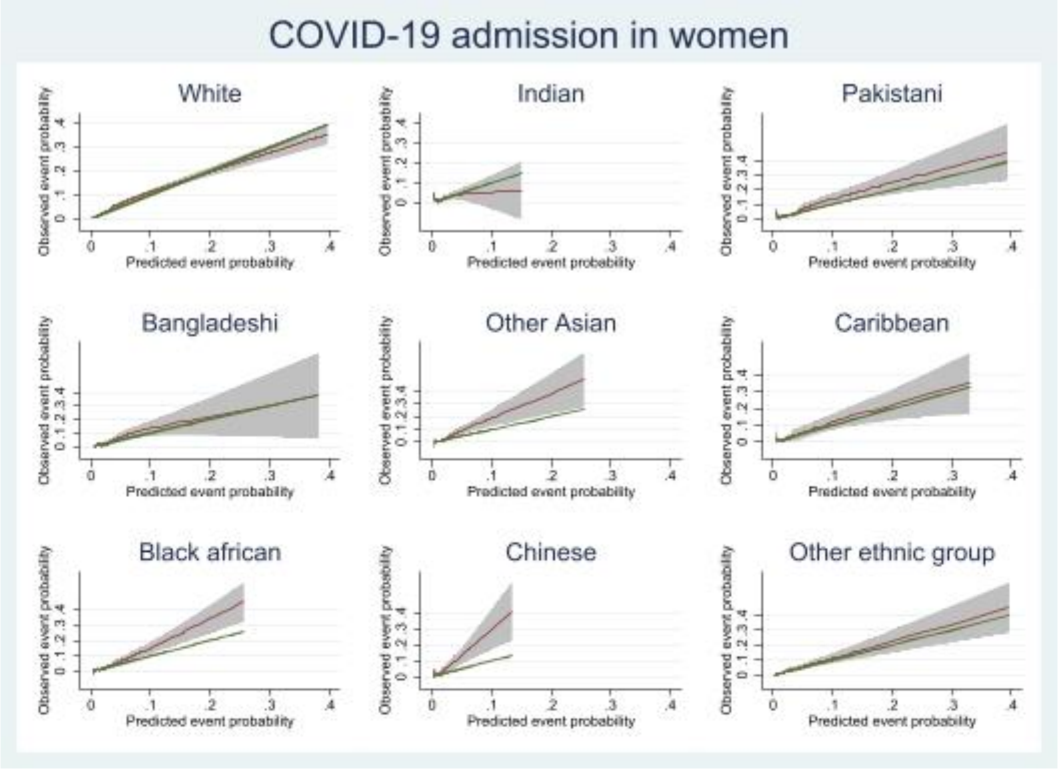

Supplementary Figure 2d Smoothed calibration plots for risk of admission by ethnic group in men

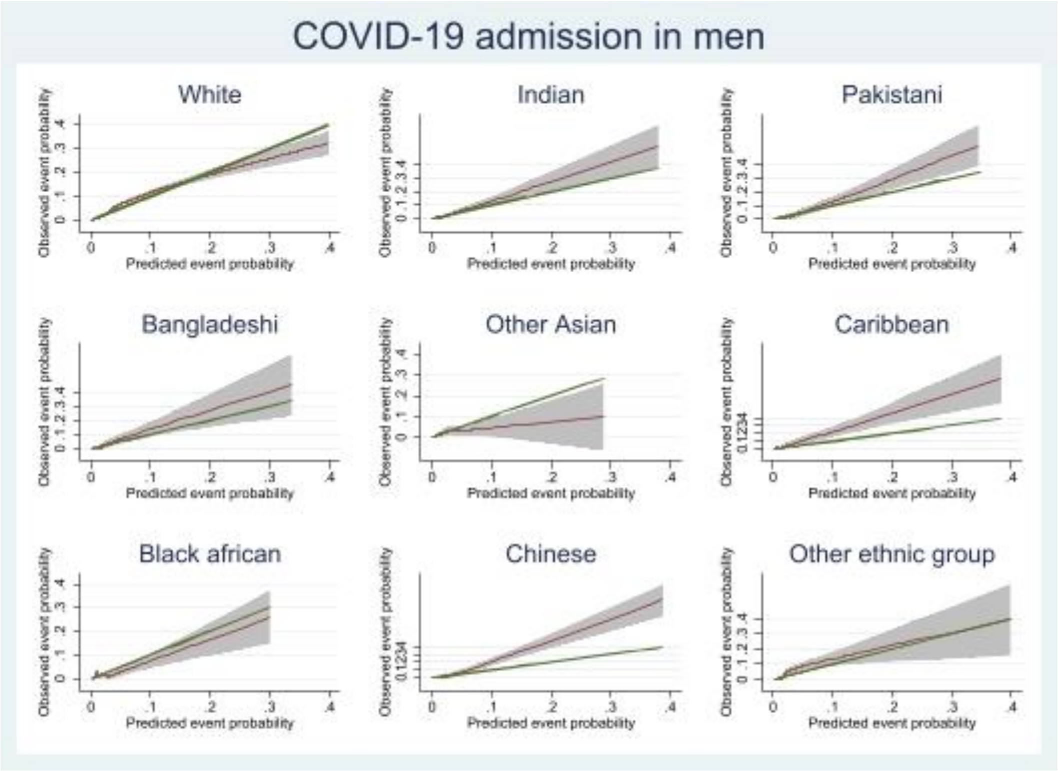

Supplementary Figure 2e Histogram of predicted risks

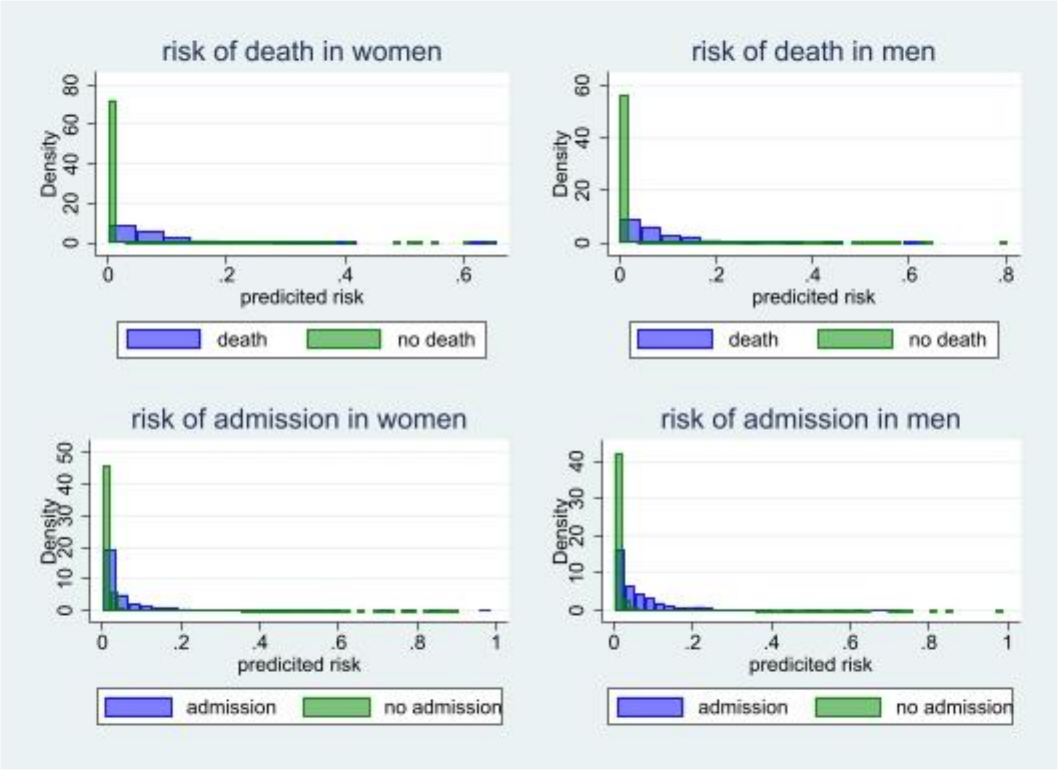

Supplementary Figure 3a Smoothed calibration plots for risk of death by region in women

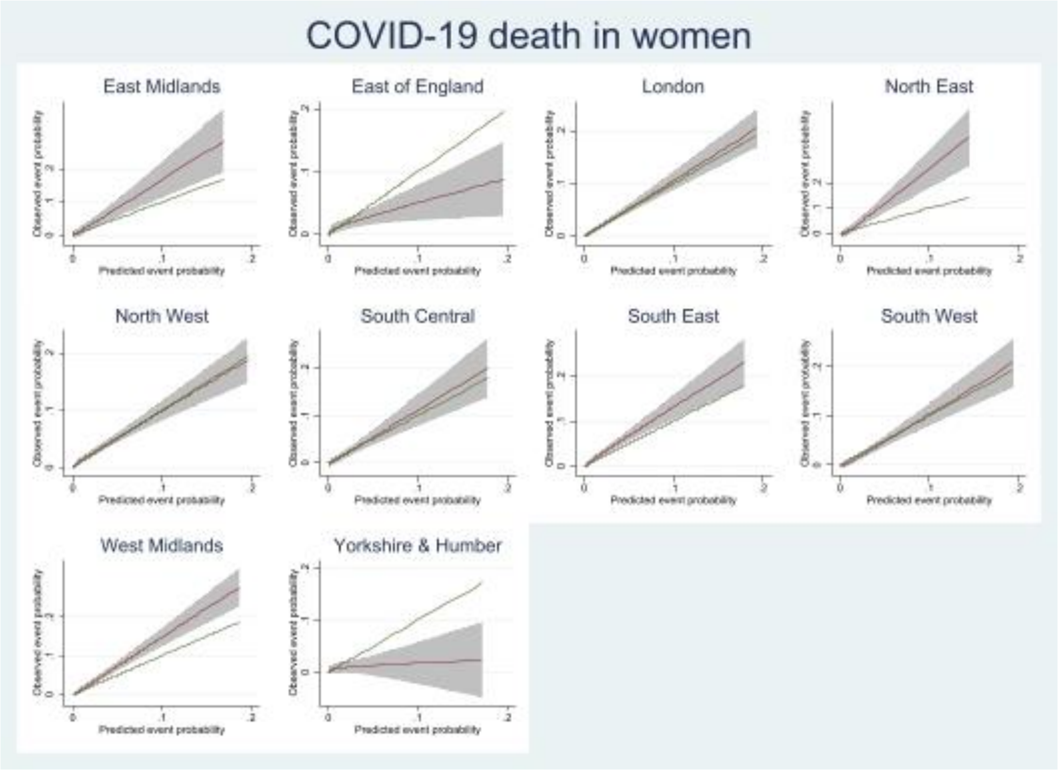

Supplementary Figure 3b Smoothed calibration plots for risk of death by region in men

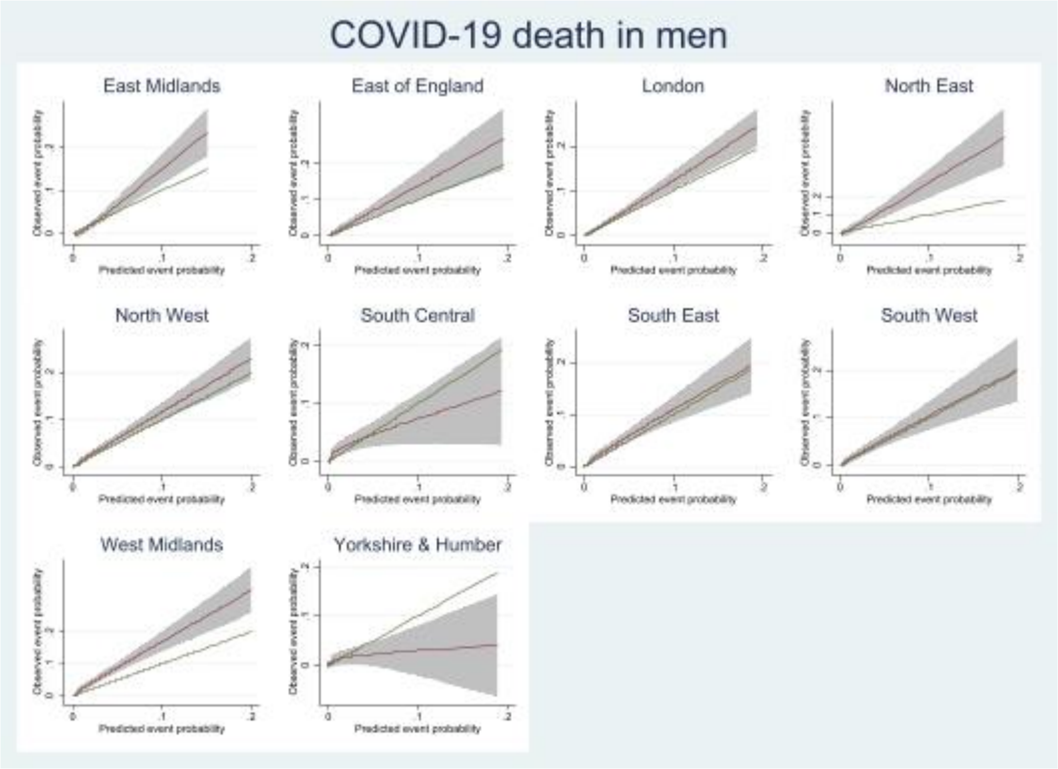

Supplementary Figure 3c Smoothed calibration plots for risk of admission by region in women

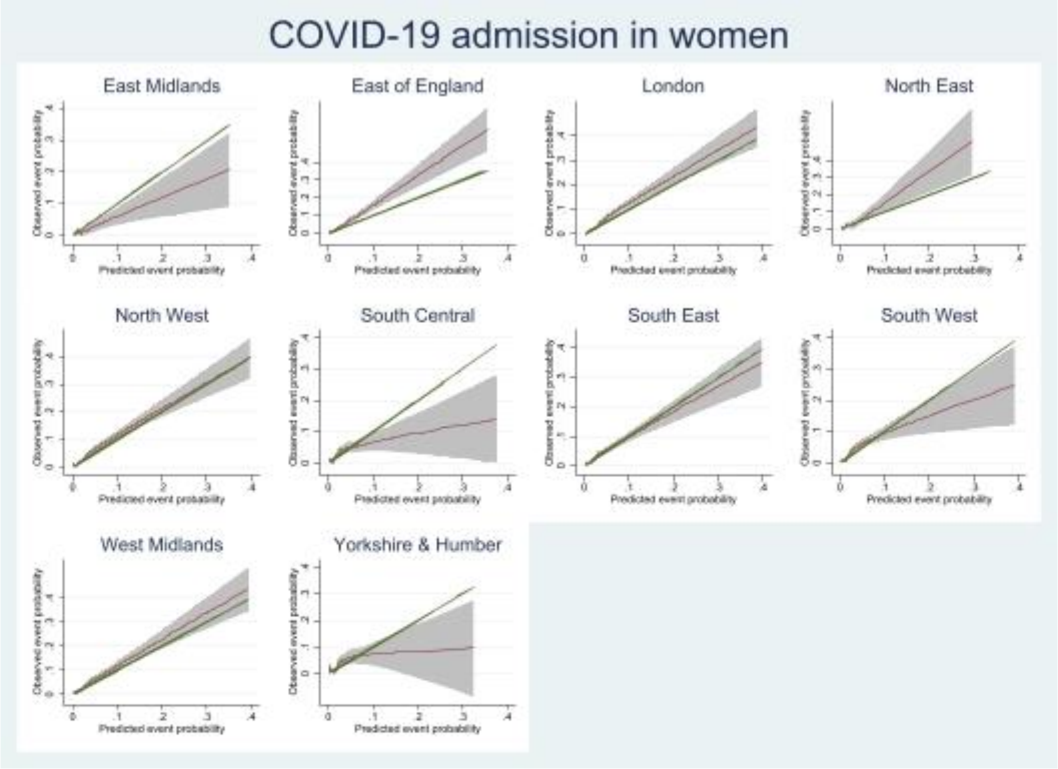

Supplementary Figure 3d Smoothed calibration plots for risk of admission by region in men

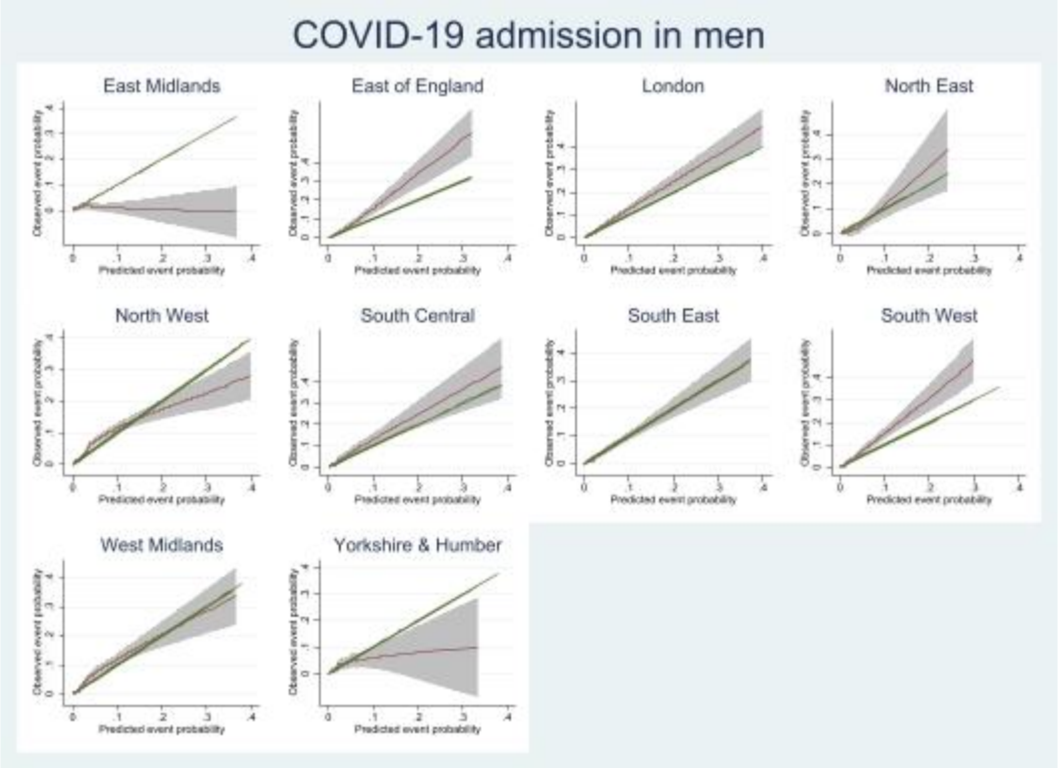

Supplementary Figure 4a Smoothed calibration plots for risk of death by age in men and women

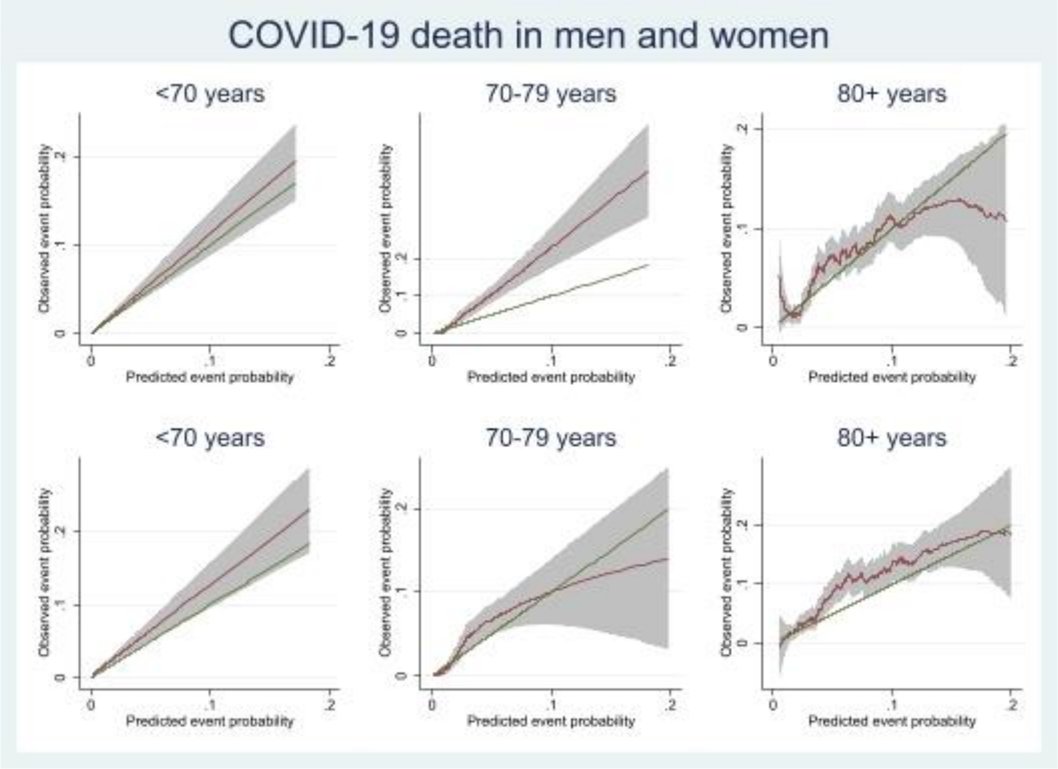

Supplementary Figure 4b Smoothed calibration plots for risk of admission by age in men and women

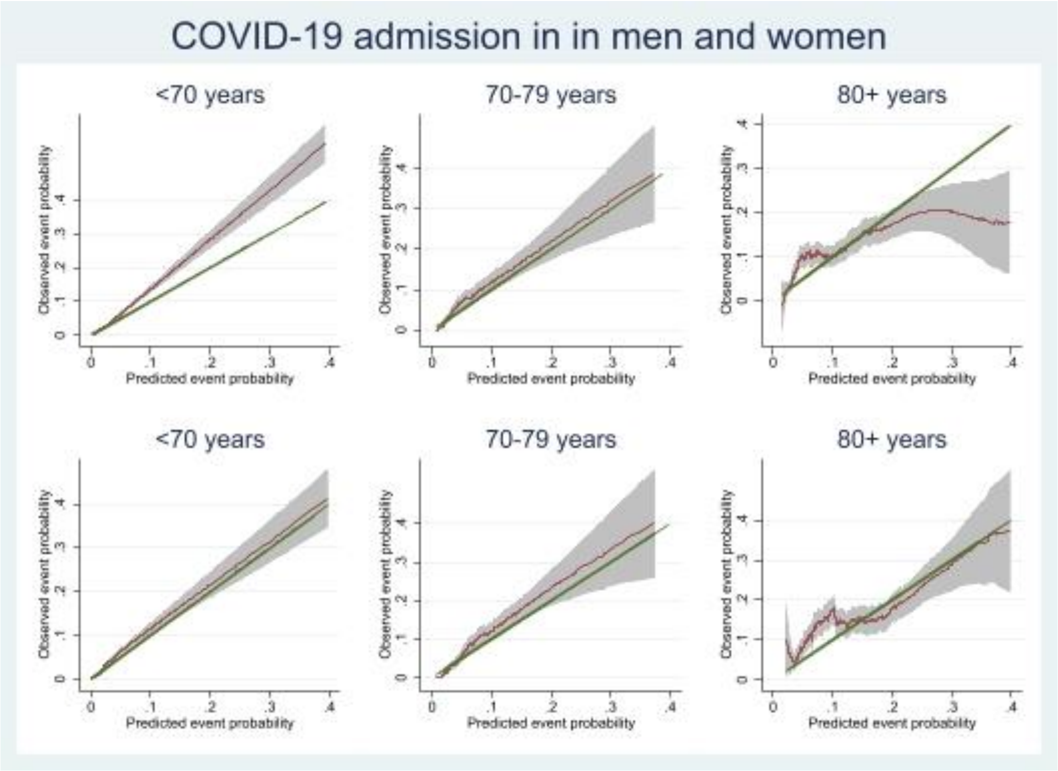

Supplementary Figure 5a Decision curve analysis for COVID-19 death using QCOVID2 and QCOVID4 by region in women

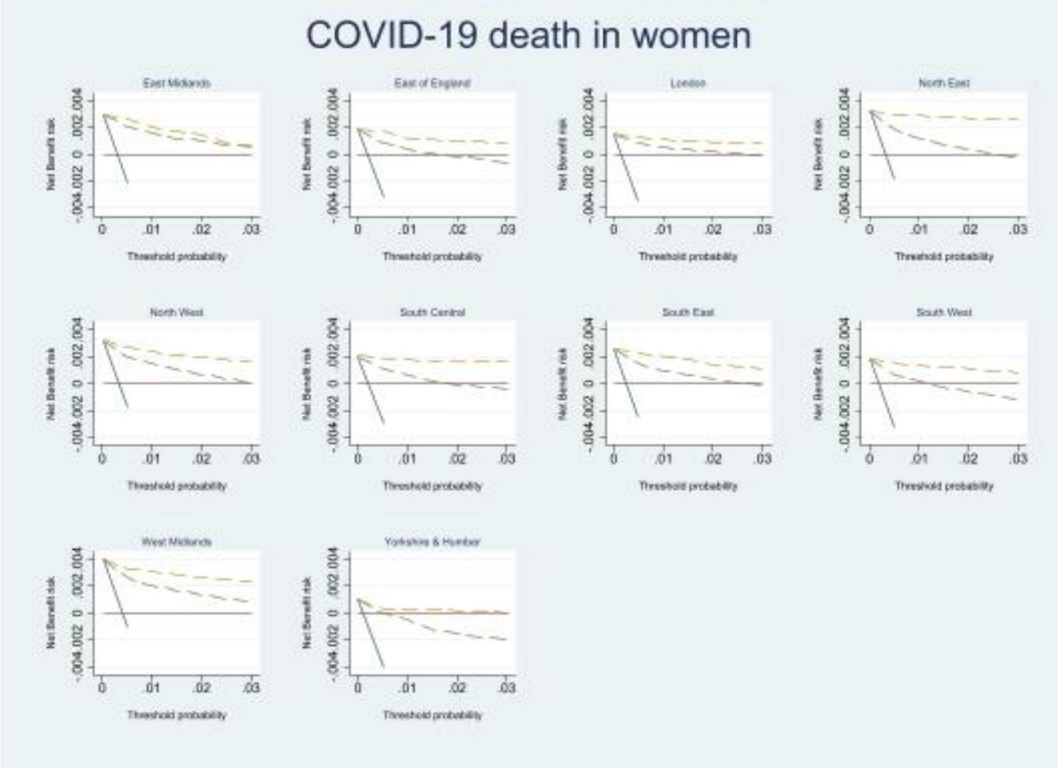

Supplementary Figure 5b Decision curve analysis for COVID-19 death using QCOVID2 and QCOVID4 by region in men

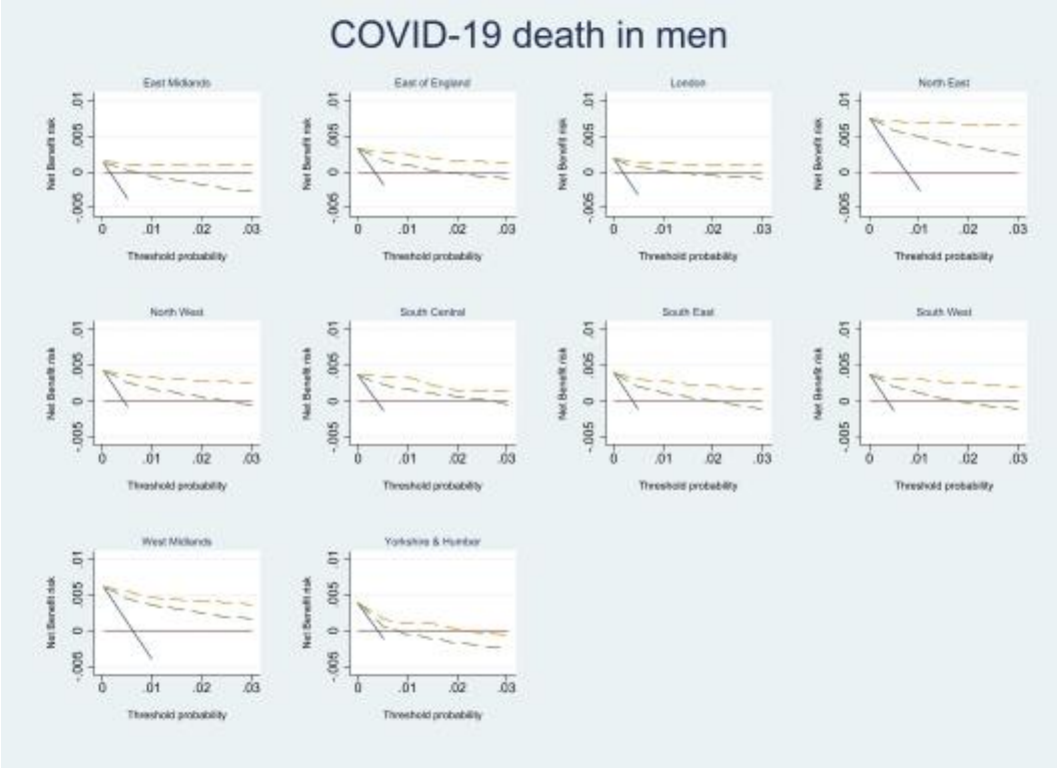

Supplementary Figure 5c Decision curve analysis for COVID-19 admission using QCOVID2 and QCOVID4 by region in women

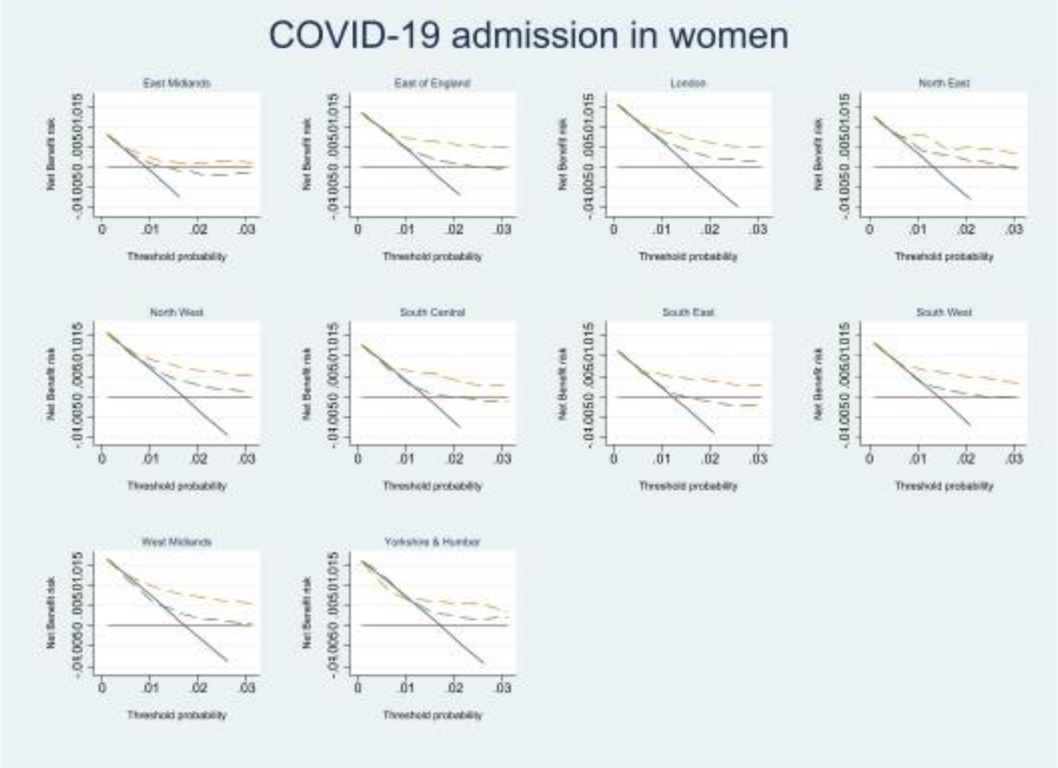

Supplementary Figure 5d Decision curve analysis for COVID-19 admission using QCOVID2 and QCOVID4 by region in men

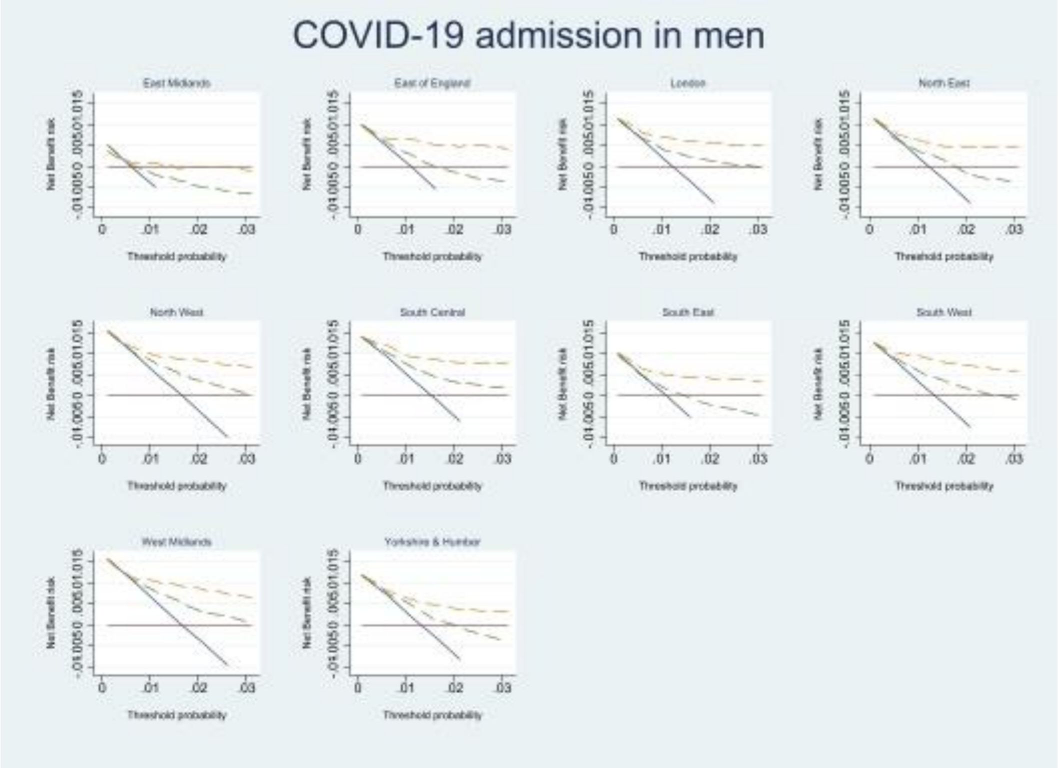

Supplementary Figure 6 Infographic

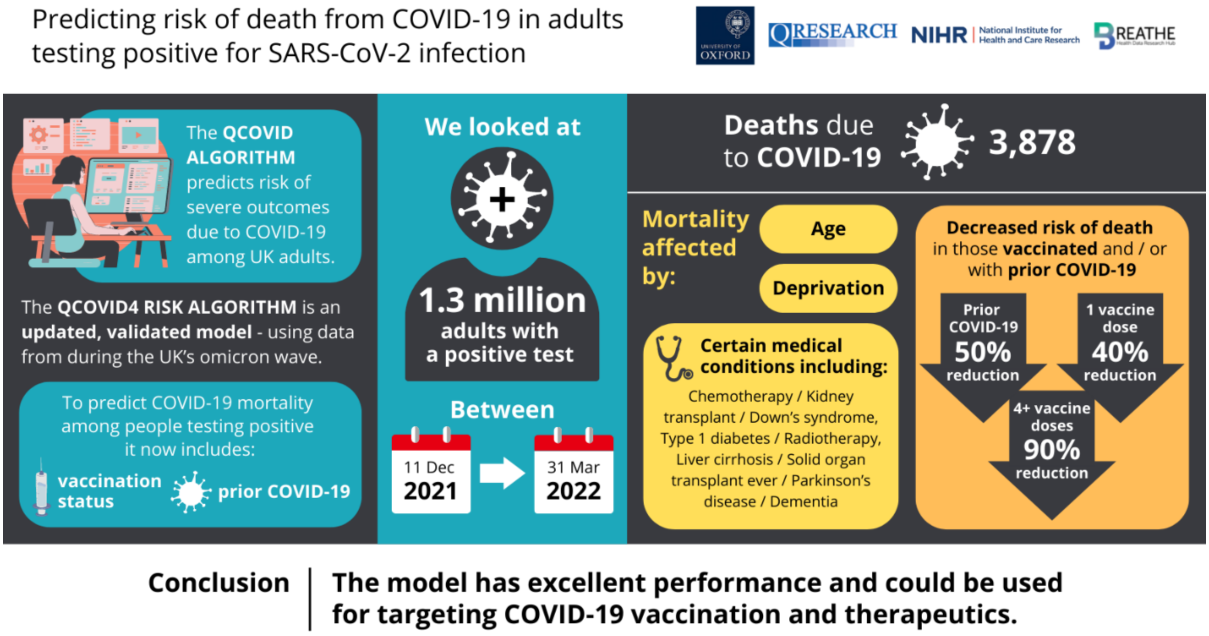

Supplement: Supplementary file 1 — Web appendix: Supplementary tables and figures [file hipj072976.ww.pdf]
